# Editing of *SlMYB60* Reveals a Role in Cuticle Formation in Tomato

**DOI:** 10.1111/ppl.70922

**Published:** 2026-05-10

**Authors:** Sara Colanero, Beatrice Landoni, Giulia Castorina, Diana Gervasoni, Manuela Maria Rigano, Silvana Francesca, Alessia Cuccurullo, Alessandro Nicolia, Aldo Sutti, Elena Baldoni, Raul Pirona, Damiano Martignago, Lucio Conti, Massimo Galbiati

**Affiliations:** ^1^ Department of Biosciences University of Milano Milan Italy; ^2^ Council for Agricultural Research and Economics (CREA), research Centre for Genomics & Bioinformatics Fiorenzuola d'Arda Italy; ^3^ National Research Council, Institute of Agricultural Biology and Biotechnology (CNR‐IBBA) Milan Italy; ^4^ Department of Agricultural Sciences University of Naples Federico II Naples Italy; ^5^ Council for Agricultural Research and Economics, Research Centre for Vegetable and Ornamental Crops Pontecagnano Italy

**Keywords:** cuticle, gene editing, MYB60, stomata, tomato

## Abstract

Under climate change, yield stability depends heavily on the ability to develop resilient crops, better adapted to water scarcity. Studies in model systems have uncovered molecular pathways and genes that potentiate the plant response to environmental stress. CRISPR‐based editing technologies enable the precise and rapid transfer of beneficial traits from model species to crops. Previous work identified *Solanum lycopersicon MYB60 (SlMYB60*) as the functional ortholog of the Arabidopsis guard cell‐related *AtMYB60* transcription factor. Loss of *AtMYB60* function results in reduced stomatal opening and enhanced stress resistance, providing a valuable target for crop improvement. Here, we report the CRISPR‐mediated exploitation of *SlMYB60* in two tomato commercial varieties. Unexpectedly, editing of *SlMYB60* did not result in reduced stomatal opening and enhanced stress resistance. Independent edited lines showed increased stomatal size, enhanced leaf water loss, and cuticle permeability. RNAseq analyses revealed that the expression of genes involved in cell wall and cuticle metabolism was altered in the edited lines. Scanning electron microscope analysis of leaf epidermis revealed defects in cuticle deposition and in the formation of outer cuticle ledges. As opposed to the guard cell‐specific activity of the *AtMYB60* promoter, we found that the *SlMYB60* promoter was active in both stomata and epidermal cells. Our findings indicate functional divergence between *AtMYB60* and *SlMYB60*, providing valuable insights into species‐specific regulatory mechanisms and emphasizing the complexities of translating gene‐editing strategies across plant systems.

## 1 Introduction

Two key epidermal features, cuticle and stomata, are at the forefront of the interplay between plants and the environment. The cuticle is a protective lipidic layer composed of cutin polyesters and waxes that cover the aerial parts of plants to prevent water loss (Arya et al. 2021). The continuity of the waxy cuticle is interrupted by microscopic valves, the stomata, formed by two highly specialized guard cells, which allow gas exchange between the plant and the atmosphere (Wang and Chang 2024). The opening and closing of the stomatal pore are mediated by turgor‐driven volume changes in two surrounding guard cells, which integrate internal signals and external cues to adapt stomatal opening to the prevailing environmental conditions (MacRobbie 1998).

Upon drought perception, the rapid closure of stomata limits water loss, preventing the dehydration of the underlying tissues (Gupta et al. 2020). Evidence indicates that enhancing stomatal activity can improve water productivity and drought resistance in crops, with limited cost to carbon fixation and biomass production (Horaruang et al. 2022). The complex network of signaling pathways that fine‐tune the opening and closing of stomatal pores has been extensively investigated in the model system *Arabidopsis thaliana* (Kim et al. 2010). These studies have highlighted that gain‐ or loss‐of‐function mutations in genes involved in stomatal regulation can significantly affect water use efficiency and stress tolerance. The recent development of gene editing technologies has opened unprecedented opportunities to do the same in crops (Dong 2024). Based on sequence similarity, putative orthologs of Arabidopsis guard cell‐related genes have been identified in many plant species, including transcription factors (TF) that specifically regulate stomatal movements (Cominelli et al. 2010).

MYB TFs is one of the largest gene families in plants. The distinctive feature of MYB proteins is the presence of a highly conserved MYB‐DNA‐binding‐domain, constituted by approximately 50 amino acids which form a helix–turn–helix (HTH) fold (Kanei‐Ishiis et al. 1990). Most plant MYBs belong to the R2R3‐type subfamily, characterized by two MYB repeats. Protein regions outside the MYB domain are highly variable and contribute to specific protein functions (Millard et al. 2019). Given the large number, the diverse spatial and temporal regulation of gene expression, MYB TFs are implicated in a variety of biological functions, including cell differentiation, development, primary and secondary metabolism, stress responses, stomatal activity, cell wall and cuticle deposition (Hwang et al. 2025).

Tomato *SlMYB60* (Solyc10g081490.1) was recently described as the functional homolog of the Arabidopsis *AtMYB60* gene, a key transcriptional integrator of guard cell responses to light, ABA, and water stress (Rodríguez‐Hoces de la Guardia et al. 2021). Inactivating *AtMYB60* in the *atmyb60‐1* mutant reduces stomatal opening and enhances stress tolerance in Arabidopsis (Cominelli et al. 2005; Simeoni et al. 2022).

The Arabidopsis and tomato MYB60 proteins share 64.4% amino acid identity, and the ectopic expression of *SlMYB60* in the *atmyb60‐1* background rescues the defective stomatal phenotype. Based on these results, *SlMYB60* was anticipated to recapitulate the *AtMYB60* function in guard cells, providing a potential target for harnessing stress adaptation in tomato (Rodríguez‐Hoces de la Guardia et al. 2021).

In this work, we report the CRISPR‐Cas9‐mediated editing of *SlMYB60* in two commercial tomato cultivars. Unlike Arabidopsis, the loss of *SlMYB60* function did not result in reduced stomatal opening and increased drought resistance in tomato. Analysis of independent editing events revealed a broad role for *SlMYB60* in regulating epidermal features, including cuticle deposition and the formation of outer cuticular ledges (OCLs). Most importantly, the edited lines did not demonstrate any advantage in terms of plant response to drought. These findings indicate divergent roles for *AtMYB60* and *SlMYB60* in Arabidopsis and tomato, respectively, and highlight the complexities of transferring gene editing strategies from model systems to crops.

## 2 Materials and Methods

### 2.1 Plant Material and Growth Conditions

This study employed two *Solanum lycopersicum* commercial cultivars, Red Setter (RS) and Ailsa Craig (AC). The two genotypes were chosen based on their different growth habits, which are known to affect water use efficiency and drought responses (Vicente et al. 2015). RS is a highly productive processing variety with a determinate growth, whereas AC is a medium‐sized fresh‐market indeterminate variety. Seeds were germinated in Jiffy pellets (Jiffy Group). Plants were transferred to 10 cm pots filled with potting soil and grown in a greenhouse in Milan (Italy) during November 2024. Light was a mix of sunlight and LED lights for 16 h/day. Physiological measurements were performed on 4‐week‐old plants with 24 pots distributed across trays (6 plants/tray). The wild type (Red Setter) had 9 replicates, the *slmyb60Cr1* line had 7 replicates, and the *Cr2* line had 8 replicates (see below about the obtention of those lines). Genotype replicates were equally distributed across trays, when possible (2 replicates/genotype/tray), and genotypes were randomized within trays. A portable Porometer/Fluorometer LI‐600 (LI‐COR) instrument was used to collect physiological (stomatal conductance to water, leaf temperature) and photosynthetic parameters (quantum yield of photosystem II—*Φ*_PSII_), as well as ambient light intensity and air temperature. All plants were measured over 1 day in the light phase, in the morning (10:00 a.m.), midday (2:00 p.m.), and the afternoon (5:00 p.m.). The measurements were taken on the first and second fully expanded leaves of each plant, one measurement per leaf. On average, based on these measurements, plants were exposed to temperatures between 14°C and 21°C and to light intensities between 170 and 210 μmol m^−2^ s^−1^ across the three sessions. Material for RNA extraction was collected from six‐week‐old plants. Samples derived by pooling four‐leaf discs/genotype from fully expanded leaves.

Another greenhouse experiment was performed in Pontecagnano (Italy) in September 2023. For the water deficit experiment, two‐week‐old seedlings were transplanted into plastic pots (11 cm diameter, 9 cm height) with commercial substrate and vermiculite (3:1 v/v). Seedlings were grown under greenhouse conditions, with 25°C ± 3°C air temperature during the day and 16°C ± 3°C during the night, and irrigated by automated drip irrigation (one 4 L h^−1^ emitter per pot). Water deficit was imposed at the three‐true‐leaf stage, defined as the point at which the third true leaf had fully expanded and the leaf area had stabilized, by suspending irrigation for 14 days. Ten plants per genotype were used for each of the tested conditions, with the pots kept covered with plastic foil to prevent evaporation from the soil. Leaf area was measured after the scanning procedure using the image analyser ImageJ (Rueden et al. 2017). Individual leaves were taken from six plants in each treatment. Relative water content was determined as follows: individual leaves from three plants per genotype per treatment were weighed (FW), saturated with distilled water for 4 h at room temperature, blotted on filter paper, and reweighed (turgid weight—TW). The dry weight (DW) was measured by drying the leaf at 80°C for 24 h. The RWC was calculated as RWC (%) = [(FW—DW)/(TW—DW)] × 100. Total biomass values were obtained at the end of treatment by weighing the shoots.

### 2.2 CRISPR/Cas9 Design, Tomato Plant Transformation, and Genotyping

Four guide RNAs (gRNA1‐4) targeting *SlMYB60* (Solyc10g081490.1) were designed using the ChopChop CRISPR web tool (https://chopchop.cbu.uib.no/) and chosen based on lower off‐target probability and greater efficiency (Table S1). The four gRNAs were assembled into the pDirect_22C plasmid through the Golden Gate cloning system, as previously described (Čermák et al. 2017). The final binary vector was introduced into *Agrobacterium tumefaciens* (strain GV3101). Tomato transformation using explants from cotyledons was carried out according to Zuluaga et al. (2008). Regenerated T0 plants were selected with kanamycin and further screened by PCR for the presence of the *Cas9* sequence with oligos sk46‐sk32. Deletions in the *SlMYB60* gene were detected by PCR using primers sk47‐sk48 and verified by direct sequencing of the PCR products obtained with primers sk3‐sk4. In this study, we used T2‐Cas9‐free homozygous *slmyb60Cr1* and ‐*Cr2* lines (RS) and bi‐allelic mutant line *slmyb60Cr3/4* (AC). Oligonucleotide sequences are described in Table S1.

### 2.3 Agroinfiltration‐Based Transient GUS Expression in *N. benthamiana*

Leaves from 5‐week‐old *Nicotiana benthamiana* plants were Agro‐infiltrated as previously described (Li 2011). In total, five leaves from four different plants were Agro‐infiltrated with the *SlMYB60*_*pro*_::*GUS:GFP* or *AtMYB60*_*pro*_::*GUS:GFP* constructs. An Arabidopsis 1348 bp region upstream of the *AtMYB60* ATG or a tomato 1480 bp sequence upstream of *SlMYB60* was amplified with primers p60GTWF1 and p60GTWR1 or primers SlMYB60F1 and SlMYB60R1 (Table S1) and cloned into the Gateway binary vector pBGWFS7 (Karimi Mansour 2002), to produce the two vectors.

Leaf samples were collected at 48 h after the Agroinfiltration and assayed for GUS activity. GUS staining was performed by vacuum‐infiltrating plant tissues in the staining solution (50 mM sodium phosphate buffer, pH 7, 0.1% Triton‐X100, 0.5 mg ml^−1^ X‐glucoronic acid, and 0.5 mM FeCN) and incubated at 37°C for 24 h. The entire leaf was cleared with 70% ethanol for 1 day. Three leaf parts corresponding with the agroinfiltrated area were placed on a slide for imaging on a S60 NanoZoomer digital slide scanner (Hamamatsu Photonics K.K.). GFP signal was detected using an A1R confocal microscope (Nikon, emission 509 nm, excitation 488 nm), and images were generated using the NIS‐Elements software.

### 2.4 Stable *SlMYB60pro* :: *GUS:GFP* Expression of Arabidopsis

Stable transgenic Arabidopsis lines (Col‐0, NASC N1092) were produced by Agrobacterium‐mediated transformation (strain GV3101) as described (Clough and Bent 1998). *SlMYB60pro*::*GUS:GFP* T2 plants were selected in vitro, in the presence of the herbicide BASTA 200 (Bayer), and used for GUS analyses. Seeds were surface‐sterilized with 100% ethanol followed by 1% NaClO, washed with sterile water, and plated on 1.5% sucrose MS medium (0.8% agar, pH 5.8). Plates were stratified at 4°C for 4 days in the dark before being transferred into the growth room at 22°C under long‐day conditions (16 h light/8 h dark). GUS staining was performed by vacuum‐infiltrating plant tissues in the GUS staining solution and incubating at 37°C for 24 h. Tissues were cleared with 70% ethanol and imaged using an SZX12 stereomicroscope (Olympus).

### 2.5 Stomatal Analyses

Stomatal opening assays were performed on epidermal strips obtained from dark‐adapted tomato leaves. Tissues were incubated in 30 mM KCl, 10 mM MES‐KOH, pH 6.5, at 22°C under white light (200 μmol m^−2^ s^−1^) for 4 h. Stomata were imaged with an Axioscope 10 microscope (Zeiss) fitted with a digital camera and analyzed with the ImageJ software (Rueden et al. 2017). Stomatal opening was expressed as the width/length ratio of the stomatal pore.

Imaging of abaxial epidermal strips was also employed for the analysis of stomatal size (SS), density (SD), and index (SI). SS was calculated as the area encompassing the stomatal pore and the two surrounding guard cells. SD was expressed as the number of stomata per unit area. SI was calculated as: (Number of Stomata × 100)/(Number of Epidermal Cells + Number of Stomata).

### 2.6 Chlorophyll Leaching and Toluidine Blue (TB) Staining Assays

The chlorophyll leaching assay was performed on mutant and wild‐type fully expanded terminal leaflets of the third compound leaves. Leaf samples were weighed, dipped in 80% (v/v) ethanol, and incubated in the dark at room temperature, up to 48 h. Chlorophyll released was quantified by measuring the absorbance at 647 nm and 664 nm with a spectrophotometer until chlorophyll extraction was complete. The concentration of chlorophyll was calculated as follows: total micromoles of chlorophyll = 7.93 × A664 + 19.53 × A647. Values were normalized per gram of FW and expressed as a percentage of total chlorophyll. TB staining was performed according to Tanaka et al. (2004). Five‐day‐old etiolated cotyledons were stained in the dark for 5 min in a 0.05% (w/v) TB and 0.1% (v/v) Tween 20 solution and rinsed five times in distilled water. For quantification, excised cotyledons were incubated in 80% ethanol for 48 h in the dark. The absorbance of the solution was measured at 626 and 430 nm using a spectrophotometer (Infinite200PRO Microplate Reader), and the total amount of TB was calculated as the A626/A430 ratio. Four biological replicates, each consisting of four cotyledons, were assessed.

### 2.7 Water Loss Measurement

To determine the rate of leaf water loss, individual leaves were detached from six plants per genotype and weighed to determine the initial FW. Sample weights were then estimated at designated time intervals, and water loss was calculated as the percentage of the initial FW.

### 2.8 SEM Analysis

For the scanning electron microscope (SEM) analysis of the epicuticular waxes, terminal leaflets from the third compound leaves from the different genotypes were air‐dried and processed according to Castorina et al. (2025). Micrographs of the abaxial and adaxial leaf surfaces were acquired with a SEM‐EDS JSM‐IT500 LV electron microscope (JEOL L.t.d.).

### 2.9 RNA Extraction and Sequencing

For RNA analysis, wild type and the T2 mutant lines *slmyb60Cr1* and *slmyb60Cr2* in the RS background were used. Leaf discs were sampled from fully expanded fourth leaves of six‐week‐old plants. This material was pooled from four plants for each biological replicate. Four biological replicates were used for each genotype (wild type, *slmyb60Cr1*, and *slmyb60Cr2*). RNA was extracted using the RNeasy Plant Mini Kit (Qiagen). Libraries were prepared from poly(A)‐enriched mRNA fractions and sequenced on a NovaSeq 6000 platform using 150 bp paired‐end reads by Biomarker Technologies (BMKGENE); the adapters and low‐quality reads were filtered out.

Bioinformatic tool and settings used to trim/filter raw reads: Fastp parameter: ‐Q ‐y ‐g ‐Y 10 ‐l 100 ‐b 150 ‐B 150 q30 > = 85%. Raw data:fastq files from BCL files with index demultiplexing. Raw data, in fastq format, were initially qualitatively analyzed. A summary of RNA‐seq data for the 12 samples is provided in Table S2. On average, about 45 million reads were produced per sample. More than 98% of the sequences had a quality above Q30, corresponding to a base call accuracy of at least 99.9%. To calculate the expression level of each gene, the Salmon software (https://combine‐lab.github.io/salmon/) was used in a Linux environment, considering the ‐‐gcBias, ‐‐seqBias, ‐‐validateMappings parameters, and employing the nucleotide sequences of the transcripts as an index (version ITAG 4.0, downloaded from https://solgenomics.net/). To identify differentially expressed genes (DEGs) between wild type vs *slmyb60Cr1*, and wild type vs *slmyb60Cr2*, the quantification files (.sf) produced with Salmon were used in the DESeq2 package using the “tximport”, “readr”, “tximportData” packages. The False Discovery Rate (FDR, Benjamini–Hochberg correction to *p* value) threshold for significance was set to < 0.05. A gene was considered differentially expressed if the log_2_(Fold Change, FC) was greater than 1 or smaller than −1. The list of common DEGs between wild type vs *slmyb60Cr1*, and wild type vs *slmyb60Cr2* was obtained, and the gene ontology analysis was performed by submitting gene IDs to the AgriGOv2 webtool (https://systemsbiology.cau.edu.cn/agriGOv2/) using ITAG4.0 *Solanum lycopersicum* database version. For promoter analysis, 1000 bp upstream sequences from the start codon were retrieved using a custom Python script (considering the strandness) in a Linux environment, saved in fasta file format, and submitted to the Plant Transcription Factor Database (PlantTFDB: https://planttfdb.gao‐lab.org/) for TF binding site prediction.

### 2.10 Statistical Analysis of Phenotypic Traits

All statistical analyses were conducted in R v4.3.2 (R Core Team 2023). ANOVA for all phenotypic traits was conducted using the function lm in the stats package stats v4.3.2 (R Core Team 2023), or lmer in the lme4 package (Bates 2015) when random intercepts were present, setting contrasts to sum for categorical predictor, and in combination with the function Anova in the car package v3.1–3 using type II sum of squares (Fox 2019). Post hoc tests to assess the difference between genotypes were conducted in emmeans v1.11.2–8 (Lenth 2025). For leaf area, RWC%, and fresh weight, the ANOVA model was specified as trait ~ genotype*treatment. For PSII efficiency, the model was: trait ~ light intensity*genotype. For stomatal conductance and leaf temperature, the model was: trait ~ genotype*time of day. For the latter two traits and PSII, tray and pot were added as random intercepts. For leaf weight loss and chlorophyll leaching, the model was: trait ~ genotype*time. For weight loss, replicate leaf was added as a random intercept, while for chlorophyll leaching, replicate sample was added as a random intercept. For all other traits, the model was: trait ~ genotype. All predictors except for light intensity (continuous) were categorical, including time variables. To improve residuals, the variables “fresh weight” and “stomatal area” in cotyledons and leaves were square‐root transformed for RS. For AC, stomata opening was log‐transformed. In the case of models with interactions between genotype and other variables, if the interaction had a statistically significant effect on traits, a post hoc test was conducted to assess differences between genotypes within levels of the other variable (e.g., within timepoints). If the interaction was not statistically significant, but the genotype was, a post hoc test was conducted to assess differences between genotypes alone or across levels of other predictors. Group means were estimated with the package emmeans, which was also used to conduct post hoc tests (Lenth R. 2025). When response variables had been transformed, post hoc tests were conducted on the transformed scale; however, estimated means and confidence intervals were back‐transformed to the original scale and have been plotted in figures. All plots were produced with ggplot2 v4.0.0 and assembled in Inkscape.

## 3 Results

### 3.1 Editing of the *SlMYB60* Gene

**FIGURE 1:**
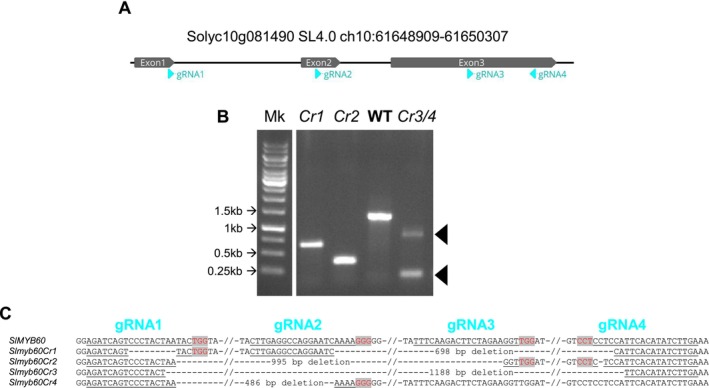
Editing of the tomato *SlMYB60* gene. (A) Structure of the Solyc10g081490 locus and localization of the guide‐RNA target sequences (light blue). (B) Amplification of the full‐length Solyc10g081490 genomic locus from two edited lines (*slmyb60Cr1* and ‐*Cr2*, cv. RS), the wild type (cv. RS), and a bi‐allelic line (*slmyb60Cr3/4*, cv. AC). (C) Genomic sequences of the *Cr1‐4* alleles, highlighting the specific CRISPR‐Cas9‐induced deletions.

To explore the role of *SlMYB60* in tomato, we obtained mutants via CRISPR‐Cas9‐mediated editing by simultaneous expression of four gRNAs, targeting the three exons of *SlMYB60*, in both Red Setter (RS) and Ailsa Craig (AC) backgrounds (Figures 1A and S1). A PCR assay on the *SlMYB60* genomic region revealed that several regenerated T0 lines, which were positive for the *Cas9* gene, carried large deletions in the *SlMYB60* amplified genomic region (Figure S1). Such deletions were identified as either a single amplicon of reduced size or two smaller amplicons, indicating that both *SlMYB60* copies were in a homozygous or heterozygous mutant state. T0 plants showing truncated *SlMYB60* versions were allowed to self, and the resulting T1 individuals were counter‐selected for the *Cas9* insertion. T2 homozygous plants inherited clear deletions in the *SlMYB60* genomic locus (Figure 1B). Two homozygote lines, *slmyb60Cr1* and *‐Cr2*, in the RS background and one biallelic line, *slmyb60Cr3/4*, in the AC background were characterized in depth. Based on the sequencing of the genomic PCR products, the mutant alleles are predicted to encode truncated proteins consisting of the first 40 amino acids of SlMYB60 (Figure 1C), followed by a variable number of amino acids before a premature stop codon. The mutant proteins displayed truncated MYB domains, composed of a partial R2 repeat and lacking the R3 repeat (Figure S1). It is thus likely that the encoded mutant alleles are non‐functional.

### 3.2 *SlMYB60* Function in Tomato Is Different From the Arabidopsis *AtMYB60* , With Respect to Guard Cell Regulation

**FIGURE 2:**
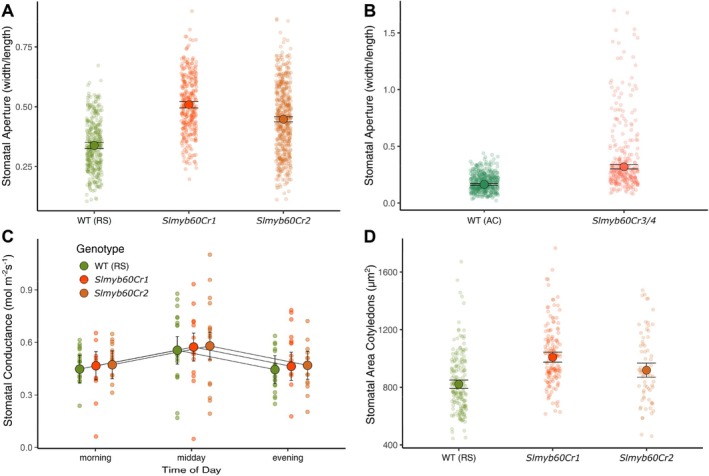
Analysis of stomatal features in the *slmyb60Cr* lines. (A) Measurement of stomatal aperture in Red Setter (RS) and in the *slmyb60Cr1* and ‐*Cr2* lines. (B) Measurement of stomatal aperture in Ailsa Craig (AC) and in the *slmyb60Cr3/4* line. Stomatal opening was assessed in epidermal strips after 4 h of exposure to white light and expressed as the width/length ratio of the stomatal pore (n RS wt = 387, *Cr1* = 343, *Cr2* = 547; n AC wt = 468, *cr3/4* = 294). Genotype had a statistically significant effect both in RS and AC background (*p* < 0.001, F RS = 173.5, F AC = 276.2) and mutants were always different from wild type according to a post hoc test (*p* < 0.001 for all comparisons; *t* RS = −18.2 (*Cr1*) and −13 (*Cr2*); *t* AC = −16.6). (C) Stomatal conductance in the *slmyb60Cr1* and ‐*Cr2* lines was measured on two different leaves per plant in the morning (10:00 a.m.), midday (2:00 p.m.), and afternoon (5:00 p.m.) with 9, 7, and 8 plants respectively measured for wild type, *Cr1*, and *Cr2* at each timepoint. Only timepoint had a statistically significant effect on stomatal conductance (*p* < 0.01, Chisq. = 13.3) but not genotype or their interaction (*p* > 0.05). (D) Measurement of stomatal size (SS) on the abaxial side of cotyledons from RS (*n* = 183), *slmyb60Cr1* (*n* = 152) and *Cr2* (*n* = 70) lines. Genotype had a statistically significant effect (*p* < 0.001, F = 33.9). Post hoc tests, wt vs. C*r1* < 0.0001 (*t* = −8.2); wt versus C*r2* < 0.01 (t‐value = −3.4). SS was calculated as the area encompassing the stomatal pore and the two surrounding guard cells. Large points represent estimated means accompanied by 95% CIs, small points represent raw data.

In Arabidopsis, the loss of the *AtMYB60* function results in impaired light‐induced stomatal opening and decreased stomatal conductance (*g*_sw_) (Cominelli et al. 2005). We investigated the effect of the *slmyb60Cr* alleles on the opening of the stomatal pores in epidermal peels excised from the leaves of wild type and edited plants and found this was affected by genotype across backgrounds according to ANOVA tests (*p* < 0.001 in both). Unexpectedly, all the *slmyb60Cr* lines displayed a slight, yet significant, increase in the opening of the stomatal pore compared with the wild type, according to post hoc tests (*p* < 0.001 for all comparisons; Figure 2A,B). Nevertheless, such an increase did not affect stomatal activity in vivo, as wild type and *slmyb60Cr* plants disclosed a comparable stomatal conductance (*g*_sw_) under standard growth conditions (*p* > 0.05; Figure 2C). Likewise, leaf temperature, collected at different diurnal time points, showed no differences compared with the wild type according to ANOVA (*p* < 0.05; Figure S2). Analyses of stomatal morphometrics revealed that genotype had a significant effect on stomatal size across backgrounds, as determined by ANOVA, in both cotyledons (Figure 2D) and leaves (Figure S3A) (*p* < 0.001 for both mutant vs. wild type comparisons). Post hoc tests showed stomatal size to be larger in the edited lines compared to the wild type for both cotyledons and leaves. Conversely, abaxial stomata density and index were not affected by genotype (*p* > 0.05; Figure S3).

**FIGURE 3:**
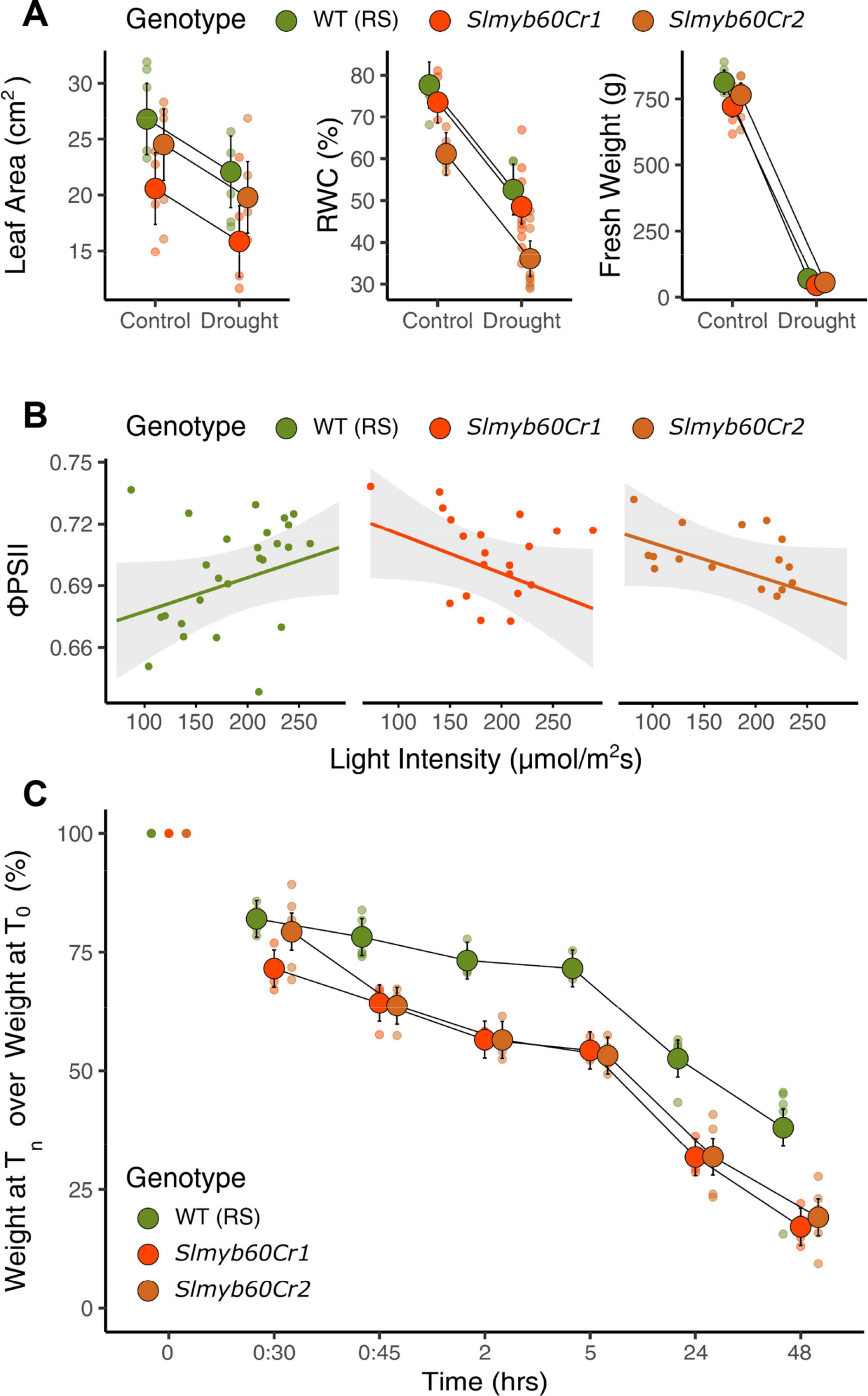
Physiological responses in RS and in the *slmyb60Cr1* and ‐*Cr2* lines. (A) Leaf Area, relative water content (RWC), and shoot fresh weight were measured 14 days after withholding of irrigation. For Leaf Area, five plants were measured for each genotype‐treatment combination. For RWC, five plants per genotype were measured under control conditions, and 3, 10, and 9 plants were measured for wild type, *slmyb60Cr1*, and ‐*Cr2* under drought, respectively. For shoot fresh weight, 6 plants were measured for each genotype under control conditions, and 3 plants under drought. Genotype and treatment, but not their interaction (*p* > 0.05), always had a statistically significant effect on leaf area (genotype: *p* < 0.05, *F* = 5.3; treatment: *p* < 0.01, *F* = 9), RWC (genotype: *p* < 0.001, *F* = 17; treatment: *p* < 0.001, *F* = 104.4), and shoot FW (genotype: *p* < 0.05, *F* = 5.1; treatment: *p* < 0.001, *F* = 2093.2). Estimated means were always lower for mutants than the wild type, but according to a post hoc test, differences were only statistically significant for: *Slmyb60Cr1* for leaf area (*p* < 0.01, *t* = 3.3); for ‐*Cr2* for RWC (*p* < 0.001, *t* = 5.1); ‐*Cr1* for fresh weight (*p* < 0.05, *t* = 3.2). Large points represent estimated means accompanied by 95% Cis; small points represent raw data. (B) Light response of the effective quantum yield of photosystem II (*Φ*_PSII_) in plants grown under control conditions. Two leaves of 9, 7, and 8 plants for wild type, *slmyb60Cr1*, and ‐*Cr2* were measured in the morning (10:00 a.m.), midday (2:00 p.m.), and afternoon (5:00 p.m.), resulting in *Φ*_PSII_ being measured at a range of light intensities for each plant. Only the interaction between light intensity and genotype had a statistically significant effect on *Φ*_PSII_ (*p* < 0.05, Chisq = 8.5), with the slope of wild type being positive and the slopes of mutants being negative, and both showing a marginal statistical difference from wild type (*p* = 0.06, *t* = 2.35). (C) Time course of water loss from detached leaves (*n* = 6 for each genotype‐timepoint combination), expressed as a percentage of the initial fresh weight at the indicated time points. Genotype (*p* < 0.001, Chisq. = 99), timepoint (*p* < 0.001, Chisq. = 2333.3), and their interaction (*p* < 0.001, Chisq. = 46.4) all had a statistically significant effect. Estimated means of mutants were lower than the wild type at all timepoints analyzed and a post hoc test showed that this difference was always statistically significant (*p* < 0.001 for all comparisons) except for the difference wild type—*Cr1* at the first timepoint (*p* > 0.05). Large points represent estimated means accompanied by 95% Cis; small points represent raw data.

To evaluate the impact of the *slmyb60Cr* mutations on whole‐plant responses to water deficit, we performed a dry‐down experiment on 5‐week‐old tomato plants (*cv* RS). Fresh weight and total leaf area were both affected by genotype (*p* < 0.05) and treatment (*p* < 0.001, *p* < 0.01, respectively), but never by their interaction (*p* > 0.05). Across conditions, the edited lines displayed a reduction in both fresh biomass and leaf area relative to the wild type, as highlighted by a post hoc test (Figure 3A). Such a trend was consistent across all comparisons, even if the decrease was not always statistically significant for some combinations of trait and edited line. Similarly, for leaf relative water content percentage (RWC%), genotype (*p* < 0.001) and treatment (p < 0.001), but not their interaction (*p* > 0.05), caused a statistically significant reduction. RWC% decreased in both mutant lines relative to the wild type, although this trend was only statistically significant for *slmyb60Cr2* (*p* < 0.001), but not for *slmyb60Cr1* (*p* > 0.05) (Figure 3A).

In an independent greenhouse experiment, measurements of quantum yield of photosystem II (*Φ*_PSII_) indicated that genotype did not affect PSII efficiency on average (*p* > 0.05). However, a statistically significant interaction between genotype and the covariate light intensity (*p* < 0.05) resulted in genotypes differing for the slope of ΦPSII (Figure 3B). A post hoc test on slopes showed that PSII efficiency increased with light intensity in the wild type but decreased in both *slmyb60Cr* lines. This could account for the reduced biomass observed in the *slmyb60Cr* plants (Figure 3A).

Detached leaves of *slmyb60Cr* mutant plants displayed a similar faster reduction in weight compared to the RS wild type (Figure 3C). Genotype, timepoint, and their interaction had a statistically significant effect on weight reduction according to ANOVA (*p* < 0.001 for all predictors), and a post hoc test showed that the percentage loss in leaf weight was significantly higher in mutants than the wild type from 45 min after detachment onwards (*p* < 0.001). This trend was also verified and significant in the *slmyb60Cr3/4* AC line (Figure S4). Collectively, these data indicate that *SlMYB60* is necessary to prevent water loss, although this may also occur through routes other than stomata.

### 3.3 *SlMYB60* Has a Broad Range of Expression and Regulates Cell Wall‐ and Cuticle‐Related Genes

**FIGURE 4:**
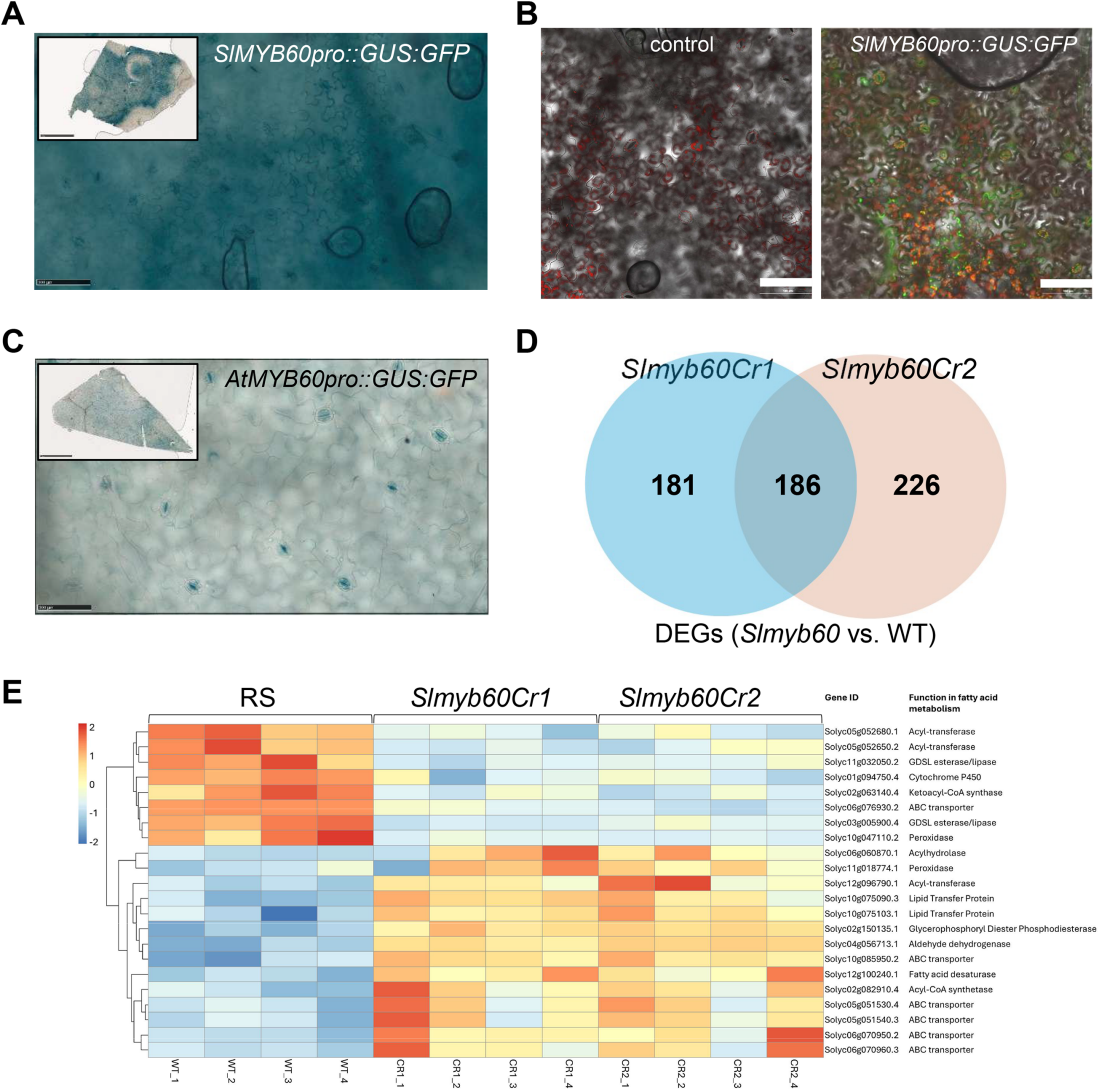
Activity of the *SlMYB60* promoter and analysis of differentially regulated genes in tomato. (A‐C) Activity of the *SlMYB60* promoter in *N. benthamiana*. Leaves were *Agro*‐infiltrated with the *SlMYB60pro:GUS:GFP* construct (A, B) or with the *AtMYB60pro:GUS:GFP* construct (C). GUS or GFP activities were investigated at 48 h after the transfection. Scale bars: 1 mm, boxed images in A and C; 100 μm in (A, B, C). (D) Venn diagram representing specific and common DEGs in the *slmyb60Cr1* and *Cr2* lines. (E) List of common DEGs encoding proteins involved in cuticle metabolism and deposition.

The detected phenotypes led us to study the patterns of expression of *SlMYB60*. Previously, we showed that a 1.3 kb *MYB60* promoter sequence of Arabidopsis and grape ( *Vitis vinifera* ) is sufficient in conferring a GC‐specific expression to the GUS reporter gene (Cominelli et al. 2011; Galbiati et al. 2011). The transcriptional fusion of a similar 1480 bp tomato promoter to GUS and GFP (*SlMYB60*_*pro*_::*GUS:GFP*) was expressed transiently in *Nicotiana benthamiana* leaves (Figure 4A,B). GUS activity was observed in the entire leaf, including GCs and epidermal cells (Figure 4A). The inspection of GFP signal via confocal imaging further confirmed the activity of the *SlMYB60*_*pro*_ in the epidermal cells (Figure 4B). The activity of the *AtMYB60* promoter from Arabidopsis using the same vector led to the expected GC‐specific GUS staining (Figure 4C). The *cis‐*organization of the *SlMYB60* promoter is similar to that of Arabidopsis, including the number and organization of DOF binding sites that are crucial for conferring GC specificity (Rusconi et al. 2013). To test whether the *SlMYB60* promoter had a GC‐specific activity in Arabidopsis, we produced stable transformants of *SlMYB60*_*pro*_::*GUS:GFP*. This resulted in a diverse range of GUS activity observed across independent transgenic lines, including the leaf vasculature, guard cells, and various other organs (Figure S5). These results collectively suggest that *SlMYB60* is not GC‐specific and regulates processes beyond stomatal activity.

To identify potential molecular pathways regulated by *SlMYB60*, we compared the transcriptome of wild type (*cv* RS) tomato leaves with that derived from the *slmyb60Cr1* and *slmyb60Cr2* lines, each represented by four biological replicates. The mRNA sequencing analysis found 367 and 412 differentially expressed genes (DEGs) in *slmyb60Cr1* and ‐*Cr2* lines, respectively, compared with the wild type (Figure 4D; Table S3). The accumulation of the *SlMYB60* transcript was significantly downregulated in the *slmyb60Cr1* and *slmyb60Cr2* lines, with a logFoldChange of −2.99 and −2.74, respectively (Table S3). Reads mapping further revealed that transcripts of *SlMYB60* in the edited lines exhibited clear truncations compared with the wild type, suggesting that partial gene products may still be produced, albeit at lower levels (Figure S6) and altered in their protein sequence (Figure S1).

DEGs common to the two mutant lines were 186, of which 146 were up‐regulated and 40 down‐regulated (Table S3; Figure S7). Among them, we identified several genes involved in cell wall metabolism. The functional classification of the common DEGs highlighted the enrichment of the Biological Process “xyloglucan metabolic process”, related to cell wall features (Figure S7). In addition, 22 common DEGs encode proteins known (or putatively expected) to be involved in cuticle metabolism and deposition (Figure 4E). These included two GDSL esterase/lipases (Solyc11g032050.2 and Solyc03g005900.4), previously shown to be involved in cuticle formation and stomata development (Shen et al. 2022), two lipid‐transfer proteins (Solyc10g075090.3 and Solyc10g075103.1), and two acyl‐CoA synthetases (Solyc02g082910.4 and Solyc02g063140.4) (Vishwanath et al. 2015). The remaining 16 transcripts (encoding ABC transporters, peroxidases, phospholipase, glycerophosphoryl diester phosphodiesterase, acyl‐transferases, aldehyde dehydrogenase, fatty acid desaturase, fatty acyl omega‐hydroxylase) are known to be involved in acyl‐lipid metabolism and were generally more expressed in the mutants (Li‐Beisson et al. 2010). The differential expression of these transcripts compared with the wild type suggested that cuticle biosynthesis might be altered in *slmyb60Cr1* and *slmyb60Cr2*.

The analysis of the promoter sequences (−1000 bp from the ATG) of the 186 common DEGs revealed an enrichment of *cis*‐elements for the binding of various TF classes, with the MYB TFs being the most represented (Figure S8). As expected, the cuticle‐related DEGs also contained putative MYB binding sites, but none of the known MYB TFs involved in cuticle formation appeared to be deregulated in the *slmyb60Cr* lines (Hwang et al. 2025).

### 3.4 SlMYB60 Regulates Cuticle Leaf Permeability

**FIGURE 5:**
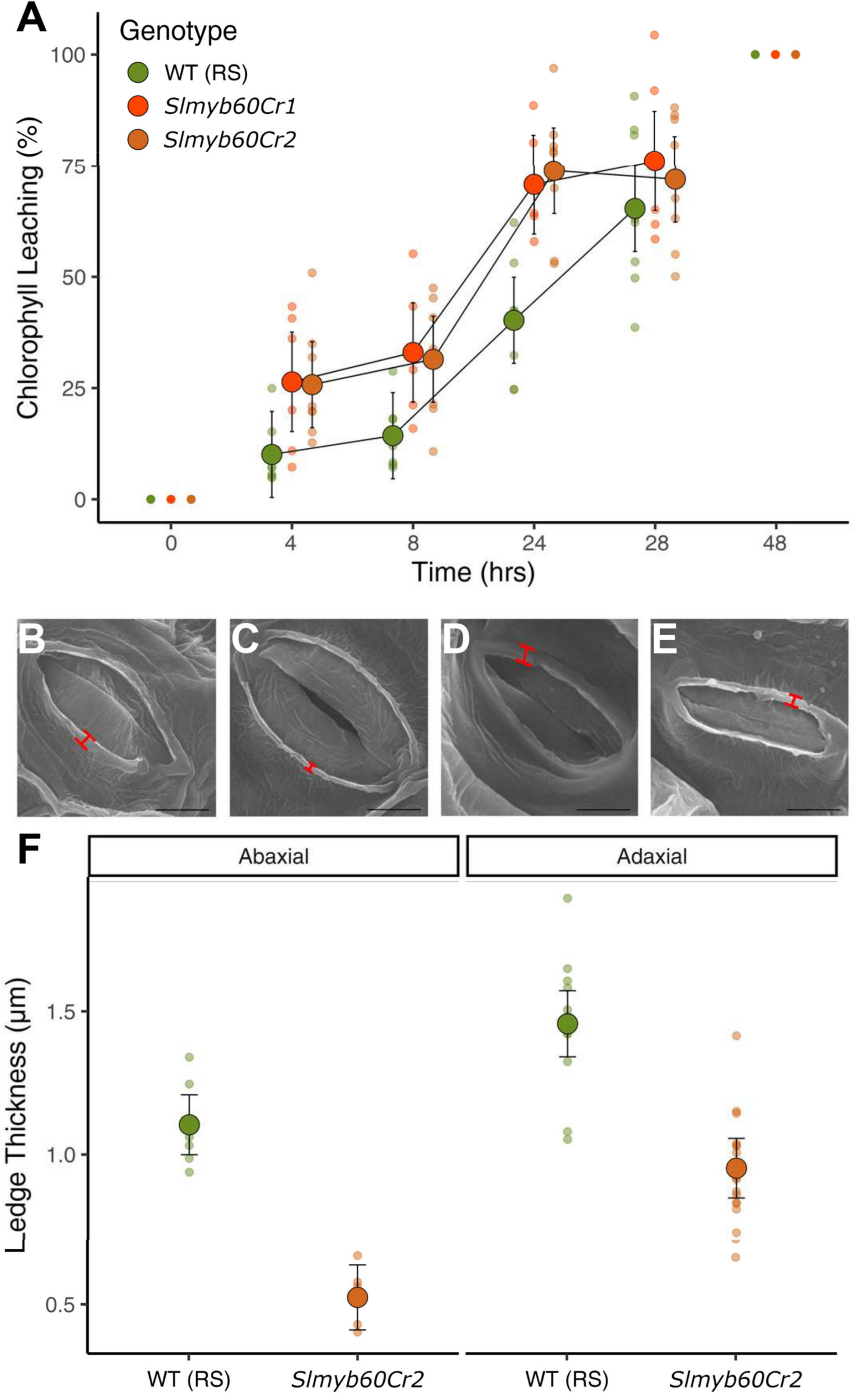
Cuticle‐related defects in *slmyb60Cr1* and *Cr2* leaves. (A) Chlorophyll leaching assay in leaves excised from wild type (*n* = 8) and edited plants (n *slmyb60Cr1* = 6, n ‐*Cr2* = 8). Genotype (*p* < 0.01, Chisq. = 13.4), timepoint (*p* < 0.001, Chisq. = 553.2), and their interaction (*p* < 0.01, Chisq. = 21.5) all had a statistically significant effect. Chlorophyll leaching was always estimated as higher in mutants than the wild type, and a post hoc showed these differences to be statistically significant at 8 (*p* < 0.05; *t Cr1* = −2.2, *t Cr2* = −2.3) and 24 h (*p* < 0.001; *t Cr1* = −4.1, *t Cr2* = −4.9). (B‐E) SEM analysis of outer cuticular ledges (OCLs). The thickness of OCLs (red lines) was measured in correspondence with stomata distributed on the abaxial (B, C) or adaxial (D, E) side of wild type (B, D) and *slmyb60Cr2* leaves (C, E). The scale bars correspond to 5 μm. (F) Quantification of OCLs thickness in the wild type and *slmyb60Cr2* line (abaxial: N WT = 7, ‐*Cr2* = 6; adaxial: N WT = 13, *Cr2* = 16). Genotype had a statistically significant effect on abaxial (*p* < 0.001, *F* = 68.7) and adaxial (*p* < 0.001, *F* = 44.3) OCL thickness, reduced in the mutant. Large points represent estimated means accompanied by 95% Cis; small points represent raw data.

Results from the RNAseq experiment pointed to a broad role of SlMYB60 in mediating epidermal features, including cuticle synthesis and deposition. We performed a chlorophyll leaching experiment to assess possible defects in the cuticle layer of leaves from the edited plants. As shown in Figure 5A, we observed a faster release of chlorophyll from *slmyb60Cr1* and *‐Cr2* mutant leaves, indicating an increased cuticle‐dependent leaf permeability compared with the wild type. Similar results were obtained for *slmyb60Cr3/4* in the AC background (Figure S9). A toluidine blue (TB) staining assay was employed on etiolated cotyledons to further investigate cuticular defects in the *slmyb60CR* lines (Tanaka et al. 2004). As expected, cotyledons from the wild type were largely impermeable to the TB dye, indicating an intact cuticular layer. On the contrary, cotyledons from the edited lines disclosed increased TB staining, confirming the presence of a defective cuticle and enhanced leaf permeability (Figure S10). These data could further explain the enhanced water loss from detached leaves, observed in the edited lines (Figure 3C).

Next, we examined the epicuticular waxes on both abaxial and adaxial leaf surfaces of wild‐type and *slmyb60* mutant plants using scanning electron microscopy (SEM). The SEM analysis revealed no appreciable differences in cuticular wax morphology between wild type and mutant leaves. This lack of divergence was likely due to the presence of an amorphous wax layer covering the leaf surface, with sparsely distributed epicuticular wax crystals (Figure S11A). Cuticle papillae, resulting from the accumulation of cuticle to form verrucous protrusions (Yang et al. 2025), were found on the surface of subulate non‐glandular trichomes (Kaur et al. 2023), yet we observed a general reduction in the density and size of the cuticle papillae on trichomes in *slmyb60Cr* mutants compared with the wild type (Figure S11B). Interestingly, *slmyb60Cr* mutants also showed significantly reduced stomatal outer cuticular ledges (OCLs) compared with the wild type, on both the abaxial and adaxial leaf surfaces (Figures 5B–F and S12). These results highlighted alterations in cuticle deposition in the *slmyb60Cr* lines, suggesting a broad role of SlMYB60 in mediating cuticle‐dependent leaf permeability in tomato.

## 4 Discussion

In this work, we provide evidence for the functional divergence of AtMYB60 and SlMYB60 in Arabidopsis and tomato. Analyses of independent edited lines indicated a broad role for the tomato SlMYB60 in mediating cuticle‐related traits in leaves, consistent with the function of other members of the SlMYB60 subgroup (Hwang et al. 2025). The tomato genome contains 127 MYB‐coding genes, whose protein products can be classified into 18 subgroups based on sequence similarity and phylogenetic topology. SlMYB60 clusters with SlMYB30, SlMYB31, SlMY94, and SlMYB96 in subgroup S7. A distinctive feature of the proteins of the subgroups is the presence of the extra‐R2R3 domain YASS[AT]ENI[AS][RK]LL[QEK][GNQ]W[MIT][KRG] (motif 11) (Li et al. 2016).

In Arabidopsis, members of the MYB subgroup 1, namely AtMYB60, AtMYB30, AtMYB31, AtMYB94, and AtMYB96, share the same conserved domain (Kranz et al. 1998). Similarities in motif patterns frequently reflect conserved protein functions (Li et al. 2016). Consistent with this hypothesis, AtMYB30, AtMYB31, AtMYB94, and AtMYB96 were found to play a role in regulating fatty acid metabolism related to cell wall and cuticle deposition (Raffaele et al. 2008; Lee et al. 2016; Shi et al. 2022). Yet, despite sequence similarity with its closest homologs, AtMYB60 exhibits a divergent cell‐specific function as a modulator of stomatal movements in Arabidopsis (Cominelli et al. 2005; Simeoni et al. 2022). One intriguing question is whether *MYB60* orthologs from different plant species retain the guard cell‐specific regulatory activity observed in Arabidopsis, or whether they play a broader role in mediating epidermal features, similarly to the other members of the *AtMYB60* clade. Evidence suggests that the cell‐specific *MYB60* function is conserved across Arabidopsis, grape, and common bean. Both the grape (*VvMYB60*) and the bean (*PvMYB60*) genes are specifically expressed in guard cells and can rescue the defective stomatal opening in the *atmyb60‐1* mutant (Galbiati et al. 2011; Simeoni et al. 2022; Martínez‐Barradas et al. 2024). Yet, in the absence of more direct functional studies, such as the CRISPR‐Cas‐mediated inactivation of *VvMYB60* and *PvMYB60* in grape and common bean, these results are far from conclusive. Interestingly, the closest *MYB60* homologs in rice (*OsMYB60*) and wheat (*TaMYB60‐1* and *TaMYB60‐2*) do not share the strong cellular specificity observed for *AtMYB60*. *OsMYB60* and *TaMYB60‐1/−2* are rapidly induced by drought stress in leaf tissues, where they primarily control leaf morphology and cuticular wax biosynthesis (Liu et al. 2018; Jian et al. 2022; Wang and Chang 2024; Zhou et al. 2024). Consistent with these findings, our work indicates a role for *SlMYB60* as a regulator of cuticular features in tomato. In this context, Arabidopsis emerges as a possible exception, as *AtMYB60* appears to have undergone functional divergence, acquiring a specialized role in the regulation of stomatal activity rather than in the modulation of cuticular deposition. According to our working model, two main features underlie the functional diversification between the Arabidopsis and tomato genes: (i) the different spatial domains of the *AtMYB60* and *SlMYB60* expression, and (ii) the different downstream targets of the two transcriptional regulators.

### 4.1 A Different Spatial Regulation for *AtMYB60* and *SlMYB60* Expression

The key feature of *AtMYB60* is the cellular specificity of its expression. Different lines of evidence, including promoter::reporter studies and laser microdissection approaches, indicate that *AtMYB60* expression is tightly confined to stomatal guard cells in Arabidopsis leaves (Cominelli et al. 2005, 2011; Galbiati et al. 2008). The transient expression of the *SlMYB60pro*::*GUS:GFP* construct in leaves of *N. benthamiana* revealed broad activity for the *SlMYB60* promoter in stomata and epidermal pavement cells (Figure 4 A‐C). Results from stable Arabidopsis lines carrying the *SlMYB60pro*::*GUS:GFP* fusion confirmed the lack of guard cell specificity for the tomato promoter (Figure S5). Interestingly, a recent comparative single‐cell expression atlas involving different plant species revealed poor conservation in the spatial regulation of guard cell‐related genes between Arabidopsis and tomato (Nguyen et al. 2025). Despite sequence similarity and conserved physiological functions, several tomato homologs of Arabidopsis genes involved in stomatal regulation are not preferentially expressed in guard cells. These include ABA signaling components, K+ uptake channels, proton pumps, and notably guard cell‐related TFs, including MYB60. By contrast, the same study revealed a high degree of conservation of the regulatory networks governing gene expression in guard cells between Arabidopsis and soybean (Nguyen et al. 2025). Differences in stomatal regulatory mechanisms between Arabidopsis and tomato are likely related to the early divergence of the two species, 150 Ma (Ku et al. 2000), as compared with the more recent divergence between Arabidopsis and soybean, which occurred nearly 90 Ma (Grant et al. 2000).

Expression of *AtMYB60* in guard cells is modulated by clusters of DOF DNA binding sequences ([A/T]AAAG), located upstream of the ATG start codon (Cominelli et al. 2011). Comparison of the Arabidopsis and tomato *MYB60* putative promoters highlighted the conservation in the distribution of DOF target motifs (Rusconi et al. 2013). It has been proposed that in Arabidopsis, expression of *AtMYB60* in most cell types is inactivated by the binding of one or more transcriptional repressors to its regulatory region (Rusconi et al. 2013). According to this hypothesis, the absence of such repressors in stomata allows binding of transcriptional activators, including the DOF protein STOMATAL CARPENTER1 (SCAP1), which promotes *AtMYB60* expression in guard cells (Negi et al. 2013; Castorina et al. 2016). We speculated that the balance between binding of transcriptional activators and repressors at the *AtMYB60* promoter is altered in *SlMYB60*. Under this hypothesis, the lack of binding sites for repressor proteins in the tomato promoter may favor the binding of transcriptional activators with diverse tissue‐ or cell‐specificity. Depending on the chromatin context, copy number, and tandem rearrangements of the *SlMYB60pro*::*GUS:GFP* insertions, this could result in preferential binding of individual TFs; therefore, conferring diverse GUS patterns as observed in the Arabidopsis transgenic lines (Figure S5). Further studies will be required to identify the *trans*‐ and *cis*‐acting elements that regulate the spatial activity of the *SlMYB60* promoter.

### 4.2 Functional Divergence of AtMYB60 and SlMYB60

Our findings indicated that *SlMYB60* inactivation did not reduce stomatal opening in tomato leaves (Figure 2A,B). One possibility is that other MYBs could compensate for the loss of *SlMYB60* and promote stomatal opening in the *slmyb60CR* lines. Notably, in Arabidopsis, the inactivation of individual MYB functions is sufficient to impair stomatal activity, suggesting a lack of functional redundancy among guard cell‐related *MYBs* (Cominelli et al. 2010). In our RNAseq analysis, we did not detect any deregulation of *MYB* genes potentially involved in regulating stomatal activity, besides *SlMYB60*. It is thus unlikely that other MYBs could compensate for the loss of the tomato *SlMYB60* function and promote stomatal opening in the *slmyb60CR* mutants. We hypothesize that the differing stomatal phenotypes resulting from the inactivation of the tomato and Arabidopsis *MYB60* genes reflect a genuine functional divergence rather than unknown compensatory mechanisms.

Evidence indicates that the Arabidopsis AtMYB60 protein plays a role in regulating oxylipin biosynthesis in guard cells. It has been proposed that AtMYB60 negatively modulates the expression of *lipoxygenase‐2*, *−4*, and *−6* (*LOX2*, *LOX4*, and *LOX6*) to reduce the accumulation of 12‐oxo‐phytodienoic acid (12‐OPDA) and jasmonate (JA), thus promoting stomatal opening (Simeoni et al. 2022). Our transcriptome analysis of wild type and *slmyb60Cr* lines did not uncover any oxylipin‐related gene among the DEGs shared by the edited lines, suggesting a possible divergence in the downstream target genes regulated by AtMYB60 and SlMYB60 (Table S3). Common DEGs between the two *slmyb60Cr* lines included genes associated with cell wall and cuticle synthesis and modification (Figure 4E). The functional classification of the common DEGs highlighted the enrichment of the Biological Process “xyloglucan metabolic process”, related to cell wall features (Figure S7). Xyloglucan is a long‐chain polysaccharide composed of a 1–4‐β‐D‐glucan backbone substituted at regular intervals with 1–6‐β‐D‐xylosyl‐residues and represents the most abundant hemicellulose in primary walls (Zablackis et al. 1995). The crosslinking of xyloglucan to cellulose microfibrils determines the subtle balance between stiffness and extensibility of cell walls, a balance that is crucial for proper stomatal activity. Mutations affecting cellulose deposition or xyloglucan synthesis are known to alter stomatal size and stomatal aperture (Rui and Anderson 2016). In this light, the increased stomatal size and the enhanced stomatal opening observed in the *slmyb60Cr* lines could be associated, at least in part, with defects in xyloglucan metabolism (Figures 2A,B,D and S3A). One hypothesis is that guard cells from the edited lines possess a less rigid cell wall, which is inefficient in counteracting the turgor pressure that inflates stomata, thus resulting in increased stomatal size and stomatal opening.

It is interesting to note that the enhanced stomatal opening observed in epidermal strips from the edited lines did not result in increased stomatal conductance in intact leaves (Figure 2C). A leading cause for the discrepancy between in vitro and in vivo measurements of stomatal activity is the viability of epidermal cells in individual strips (Spence et al. 1983). This issue could be particularly relevant in the *slmyb60Cr* lines, as defects in the cell wall could alter the mechanical interactions between stomata and surrounding epidermal cells, resulting in an exaggerated response in vitro. Transcriptomic analyses also uncovered genes involved in cuticle biogenesis among the differentially expressed genes, suggesting the possible involvement of SlMYB60 in regulating the deposition of the cuticular layer in the tomato leaf (Figure 4E). Interestingly, the expression of cuticle biosynthetic genes (e.g., GDSL esterases/lipases, acyl‐CoA synthetases) was downregulated in the *slmyb60Cr* lines compared with the wild type, whereas genes encoding transporter proteins (e.g., ABC transporters) were upregulated (Figure 4E). It is intriguing to speculate that the reduced biosynthetic activity could trigger a compensatory mechanism to offset the reduced synthesis of cuticle monomers.

None of the known MYB TFs involved in cuticle formation appeared to be deregulated in the *slmyb60Cr* lines (Hwang et al. 2025). This evidence could indicate a direct role for *SlMYB60* in controlling the expression of specific cuticle‐related genes and the lack of functional redundancy among cuticle‐related MYBs in tomato.

Consistent with results from the RNAseq experiment, the *slmyb60Cr* lines displayed a higher degree of water loss compared with the wild type, as demonstrated by increased transpiration from detached leaves (Figures 3C and S4) and enhanced cuticle‐dependent leaf permeability (Figure 5A). Cell walls and cuticle are highly integrated, with the latter being tightly anchored to the outer cell wall of epidermal cells (González‐Valenzuela et al. 2023). In correspondence with stomata, the extension of the guard cell wall initiates epidermal structures, known as outer cuticular ledges (OCLs). Proper development of OCLs is required for stomatal activity, as the tilting of their orientation helps the opening and closing of the stomatal pore. In addition, OCLs provide a mechanical barrier that prevents the loss of water when stomata are closed and the entry of water droplets when stomata are open (Zhao and Sack 1999). OCL formation is severely compromised in the above‐mentioned *gpat4 gpat8* double mutant, impaired in cutin synthesis, indicating the relevance of cutin in OCL development (Li et al. 2007). Moreover, mutations affecting OCL formation display larger stomatal size and impaired stomatal activity, compared with the wild type (Hunt et al. 2017). Notably, we observed a significant reduction in the width of OCLs in stomata from the *slmyb60Cr* mutants, on both the abaxial and adaxial leaf epidermis (Figure 5F). The impairment of OCLs formation is conceivable with the cuticular defects observed in the *slmyb60Cr* lines and likely contributes to enhancing stomatal size and stomatal opening in the edited lines. The presence of defective OCLs in the edited lines also indicates that, in addition to a broad role in regulating cell wall and cuticular features in the leaf epidermis, SlMYB60 retains a cell‐specific activity, modulating the formation of cuticular structures in stomata. In conclusion, our work provides novel insights into the diverse functional significance of the MYB60 regulatory network in Arabidopsis and tomato, unravelling unforeseen limitations for the biotechnological exploitation of *SlMYB60* to improve stress resilience in tomato.

## Author Contributions

**Massimo Galbiati** and **Lucio Conti:** conceptualization, writing of initial draft; **Damiano Martignago**, **Aldo Sutti**, and **Sara Colanero:** gRNAs design, plant transformation, analysis of editing events. **Sara Colanero, Elena Baldoni**, and **Diana Gervasoni:** physiological and molecular analyses. **Manuela Maria Rigano**, **Silvana Francesca**, **Alessia Cuccurullo**, and **Alessandro Nicolia:** greenhouse drought experiment. **Beatrice Landoni:** statistical analyses. **Giulia Castorina:** SEM analyses. **Elena Baldoni** and **Raul Pirona:** RNAseq dataset analyses. **Massimo Galbiati** and **Lucio Conti:** supervision and funding acquisition. All authors contributed to the drafts and approved the final manuscript.

## Funding

This study was funded by “European Union‐ Next Generation EU, Mission 4, Component 1 CUP B53D23032000001, project TOLERANT (P20228HKHM)” to MG and LC, and carried out within the and received funding from the European Union Next‐Generation EU (Ministero dell'Università e della Ricerca (PNRR)—MISSIONE 4 COMPONENTE 2, INVESTIMENTO 1.4‐D.D. 1032 17/06/2022, CN00000022). The generation of Crispr lines was supported by a grant from the Italian Ministry of Agriculture (MiPAAF), project BIOTECH‐Cisget, to LC.

## Disclosure

Generative AI statement: No generative AI tools were used in the generation of data, data analysis, or interpretation of the results.

## Supporting information


**Figure S1:** Editing of the tomato SlMYB60 gene. PCR amplification of Slmyb60 (top) and Cas9 (bottom) sequences from independent T0 regenerated tomato plants in either the AC or RS backgrounds. Non‐transformed AC served as the positive control, while “‐” denotes a no‐DNA PCR control. Lines shown in bold were selected for further analysis. The lower panel illustrates the predicted protein products derived from alleles slmyb60Cr1–4. Blue and green boxes represent the R2 and the R3 MYB repeats, respectively.
**Figure S2:** Leaf Temperature (°C) in wild type, slmyb60Cr1, and Cr2 lines, was measured on two different leaves per plant in the morning (10:00 a.m.), midday (2:00 p.m.), and afternoon (5:00 p.m.) with 9, 7, and 8 plants measured for wild type, Cr1, and Cr2 at each timepoint, respectively. Only timepoint had a statistically significant effect on stomatal conductance (*p* < 0.001, Chisq.‐value = 1632.9) but not genotype or their interaction (*p* > 0.05). Large points represent estimated means accompanied by 95% CIs, small points represent raw data.
**Figure S3:** Analysis of stomatal features in the abaxial side of the wild type (RS) and slmyb60Cr1 and ‐Cr2 lines. Stomatal area was assessed for *n* = 147, 112, and 154 stomata for wild type, slmyb60Cr1, and ‐Cr2, respectively. Stomatal density and stomatal index were measured by counting the number of stomata and epidermal cells on the abaxial leaf surface cleared with 80% EtOH. Five leaves were analyzed for each genotype, for a total of 20 images per line, corresponding to an overall area of approximately 5 mm2. Stomatal density was expressed as the number of stomata per mm2 of epidermal surface. Stomatal index was calculated as: (Number of Stomata × 100)/(Number of Epidermal Cells + Number of Stomata). Genotype only had a statistically significant effect on stomatal area (*p* < 0.001, *F* = 12.7), with stomata always larger in mutants than wild type and this difference being statistically significant both for slmyb60Cr1 (*p* < 0.05, *t* = −2.7) and ‐Cr2 (*p* < 0.001, *t* = −5) according to a post hoc test. Large points represent estimated means accompanied by 95% CIs, small points represent raw data.
**Figure S4:** Time course of water loss from excised leaves (*n* = 6 for each genotype‐timepoint combination) in the wild type (AC) and the slmyb60Cr3/4 line, expressed as a percentage of the initial fresh weight at the indicated time points. Genotype (*p* < 0.001, Chisq. = 12.2), timepoint (*p* < 0.001, Chisq. = 507.4), and their interaction (*p* < 0.01, Chisq. = 18.2) all had a statistically significant effect. Estimated means of mutants were lower than wild type at all timepoints analyzed, and a post hoc test showed that this difference was statistically significant at 24 (*p* < 0.001, *t* = 4.7) and 48 h (*p* < 0.001, *t* = 3.7). Large points represent estimated means accompanied by 95% CIs, small points represent raw data.
**Figure S5:** GUS expression patterns in seedlings harboring the SlMYB60pro:GUS:GFP construct. GUS assay was performed on 15‐day‐old plants. The table reports a summary of different GUS profiles observed in independent lines. −, no GUS signal; +, weak GUS signal; ++, moderate GUS signal; +++, strong GUS signal; Ep, epidermis; trich., trichomes; VASC, vasculature; GC, guard cells.
**Figure S6:** Snapshot of the Integrative Genome Viewer window software representing the mapped reads on the Slmyb60 gene (ITAG 4.0 reference) in wild type, slmyb60Cr1, and ‐Cr2 lines. Differences in spliced reads, indicated by blue (forward reads) and red (reverse reads) arcs, between the wild type and the mutants show different gene structure in the same way as exons reads coverage. At the bottom, the gene structure of Slmyb60 is shown with boxes representing the exons and line the introns. Arrows indicate the gene direction on chromosome.
**Figure S7:** Heatmap of the common DEGs between the two mutant lines. Numbers 1 to 4 represent the biological replicates. The colored scale represents the normalized expression value. At the bottom, the top‐down hierarchical graphical result of the significant gene ontology term found in the common DEGs performed on AgriGOv2 website.
**Figure S8:** Histogram of TFs present in the tomato genome reference ITAG4.0 (light blue) and the TFs found to be possibly involved in common DEGs transcriptional regulation (dark blue).
**Figure 9:** Chlorophyll leaching assay in leaves excised from wild type AC and slmyb603/4 edited plants, with *n* = 10 leaves sampled for each genotype‐timepoint combination. Genotype (*p* < 0.001, Chisq. = 30), timepoint (*p* < 0.001, Chisq. = 4296), and their interaction (*p* < 0.001, Chisq. = 90.6) all had a statistically significant effect. Chlorophyll leaching was always estimated as higher in mutants than wild type, and a post hoc showed these differences to be statistically significant at all timepoints (*p* < 0.05) except the last one. Large points represent estimated means accompanied by 95% CIs, small points represent raw data.
**Figure S10:** TB staining of wild type and slmyb60CR lines. Increased staining of the slmyb60Cr1 and −2 (A) or of the slmyb603/4 line (B) compared with the respective wild type plants (RS or AC) indicated altered permeability of the cuticle layer. Scale bar: 1 mm. (C) Quantification of the TB staining. Asterisks indicate significant differences compared with the wild type (ANOVA, *p* < 0.001).
**Figure S11:** SEM micrographs of epicuticular wax layer. Pavement cells (A) and non‐glandular trichomes (B) on the abaxial and adaxial side of the fully expanded third leaf (terminal leaflet) have been analysed in Ailsa Craig (AC) and Red setter (RS) wild‐type (WT) background, slmyb60Cr3/4, slmyb60Cr1 and slmyb60Cr2 edited plants. White arrowheads point to cuticle papilla on trichomes (B). Scale bars correspond to 5 μm in the micrographs of trichomes, and to 1 μm for the epidermis.
**Figure S12:** SEM micrographs of the stomatal outer cuticular ledges (OCLs) in the wild type (AC, RS) and in the slmyb60Cr3/4 and slmyb60Cr1 edited lines. Red lines indicate the thickness of OCLs on the abaxial and adaxial side of fully expanded third leaves (terminal leaflets). Scale bars correspond to 5 μm.
**Table S1:** List of guideRNAs and primers used in the study.
**Table S2:** Sequencing and mapping data. Total reads refer to the raw sequening output. Filtered reads refers to the number of reads after trimming. Salmon targets refer tothe number of transcripts used in the Salmon pipeline.
**Table S3:** List of common differentiallyexpressedgenes (DEGs) in the slmyb60Cr1and slmyb60Cr2lines comparedwith the wild type.

## Data Availability

The data that support the findings of this study are openly available in GEO at https://www.ncbi.nlm.nih.gov/geo/, reference number GSE319218.
