# Supplementary material for: Editing of SlMYB60 Reveals a Role in Cuticle Formation in Tomato
Source: Physiol Plant. 2026 May 10;178:e70922. doi: 10.1111/ppl.70922 (PMC13158055; doi:10.1111/ppl.70922)
Supplement: Supplementary file 1 — Figure S1: Editing of the tomato SlMYB60 gene. PCR amplification of Slmyb60 (top) and Cas9 (bottom) sequences from independent T0 regenerated tomato plants in either the AC or RS backgrounds. Non‐transformed AC served as the positive control, while “‐” denotes a no‐DNA PCR control. Lines shown in bold were selected for further analysis. The lower panel illustrates the predicted protein products derived from alleles slmyb60Cr1–4. Blue and green boxes represent the R2 and the R3 MYB repeats, respectively. Figure S2: Leaf Temperature (°C) in wild type, slmyb60Cr1, and Cr2 lines, was measured on two different leaves per plant in the morning (10:00 a.m.), midday (2:00 p.m.), and afternoon (5:00 p.m.) with 9, 7, and 8 plants measured for wild type, Cr1, and Cr2 at each timepoint, respectively. Only timepoint had a statistically significant effect on stomatal conductance (p < 0.001, Chisq.‐value = 1632.9) but not genotype or their interaction (p > 0.05). Large points represent estimated means accompanied by 95% CIs, small points represent raw data. Figure S3: Analysis of stomatal features in the abaxial side of the wild type (RS) and slmyb60Cr1 and ‐Cr2 lines. Stomatal area was assessed for n = 147, 112, and 154 stomata for wild type, slmyb60Cr1, and ‐Cr2, respectively. Stomatal density and stomatal index were measured by counting the number of stomata and epidermal cells on the abaxial leaf surface cleared with 80% EtOH. Five leaves were analyzed for each genotype, for a total of 20 images per line, corresponding to an overall area of approximately 5 mm2. Stomatal density was expressed as the number of stomata per mm2 of epidermal surface. Stomatal index was calculated as: (Number of Stomata × 100)/(Number of Epidermal Cells + Number of Stomata). Genotype only had a statistically significant effect on stomatal area (p < 0.001, F = 12.7), with stomata always larger in mutants than wild type and this difference being statistically significant both for slmyb60Cr1 (p < 0.05, [file PPL-178-e70922-s001.pdf]

Supporting Information

Editing of *SlMYB60* reveals a role in cuticle formation in tomato

Sara Colanero, Beatrice Landoni, Giulia Castorina, Diana Gervasoni, Manuela Maria Rigano, Silvana Francesca, Alessia Cuccurullo, Alessandro Nicolia, Aldo Sutti, Elena Baldoni, Raul Pirona, Damiano Martignago, Lucio Conti, Massimo Galbiati.

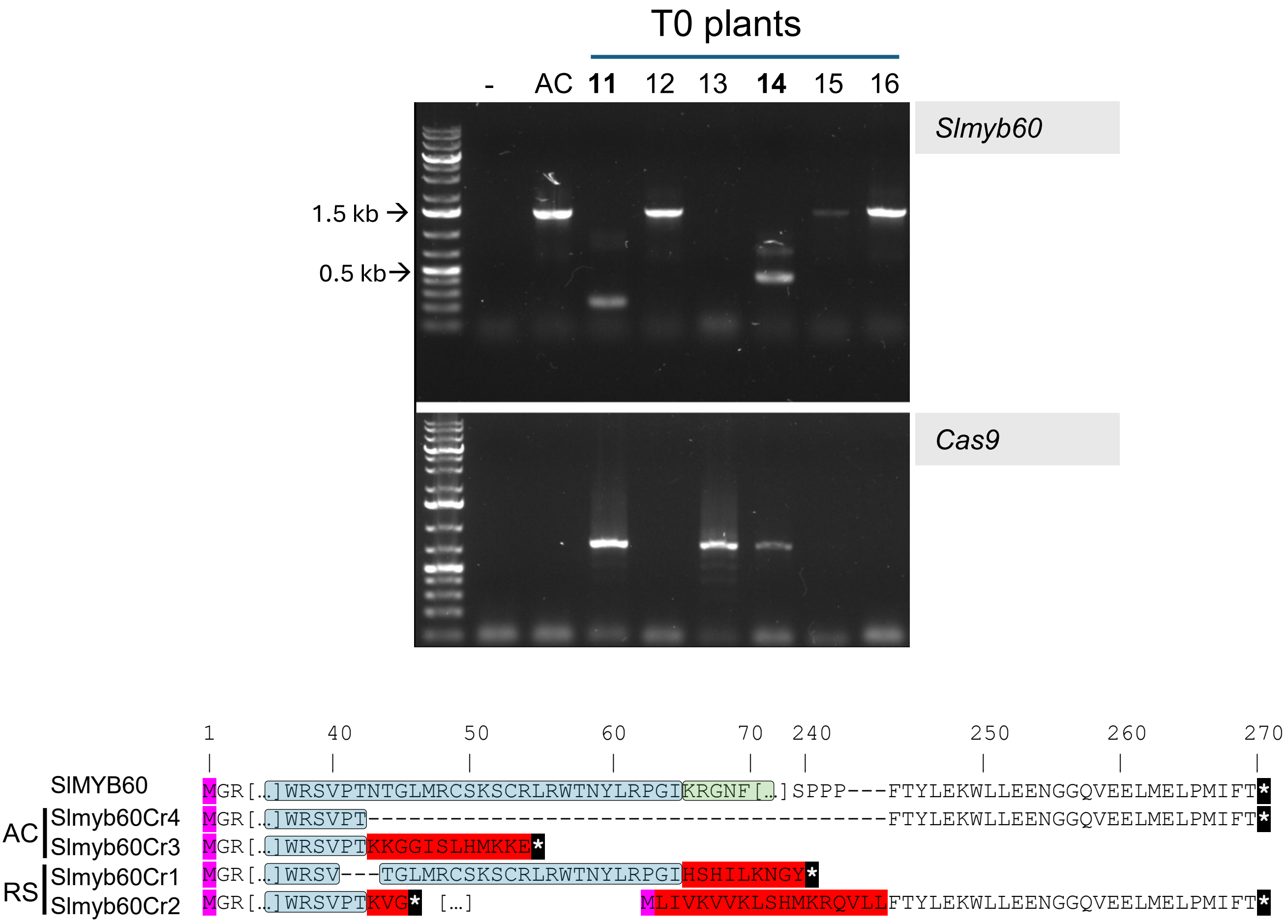

SUPPLEMENTARY FIGURE 1: Editing of the tomato *SlMYB60* gene. PCR amplification of *Slmyb60* (top) and *Cas9* (bottom) sequences from independent T0 regenerated tomato plants in either the AC or RS backgrounds. Non-transformed AC served as the positive control, while "-" denotes a no-DNA PCR control. Lines shown in bold were selected for further analysis. The lower panel illustrates the predicted protein products derived from alleles *slmyb60Cr1–4*. Blue and green boxes represent the R2 and the R3 MYB repeats, respectively.

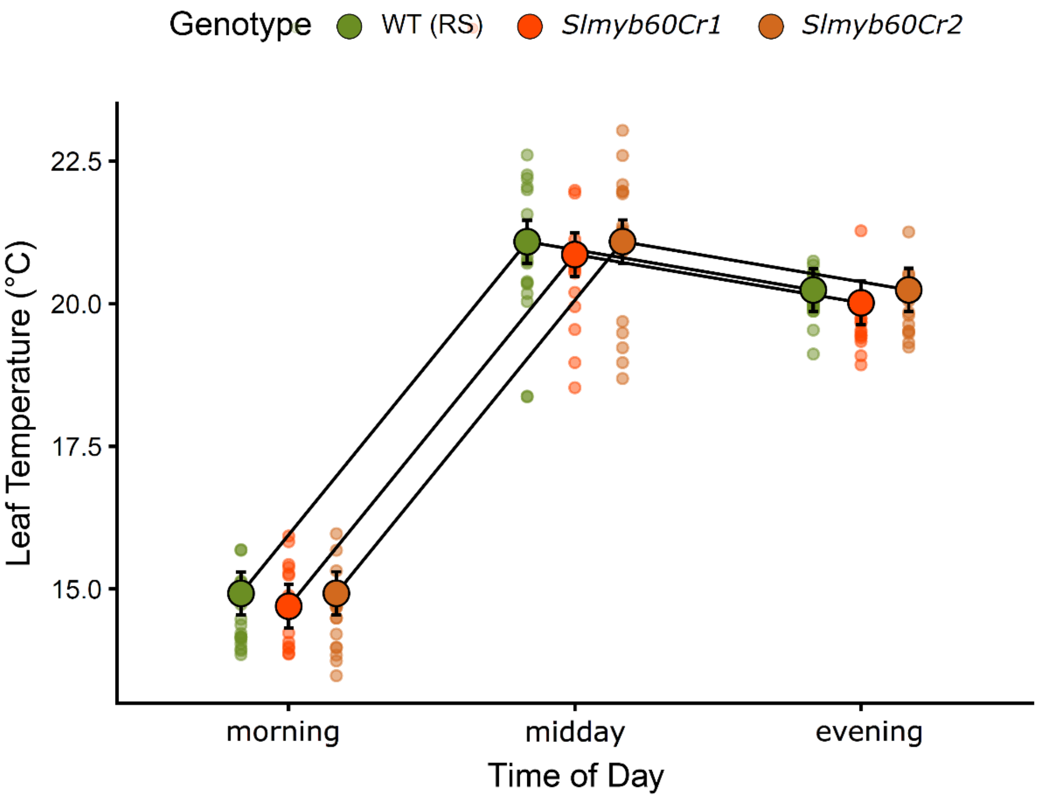

SUPPLEMENTARY FIGURE 2: Leaf Temperature (°C) in wild type, *slmyb60Cr1*, and *Cr2* lines, was measured on two different leaves per plant in the morning (10:00 a.m.), midday (2:00 p.m.), and afternoon (5:00 p.m.) with 9, 7, and 8 plants measured for wild type, *Cr1*, and *Cr2* at each timepoint, respectively . Only timepoint had a statistically significant effect on stomatal conductance ($p < 0.001$, Chisq.-value =1632.9) but not genotype or their interaction ($p > 0.05$). Large points represent estimated means accompanied by 95% CIs, small points represent raw data.

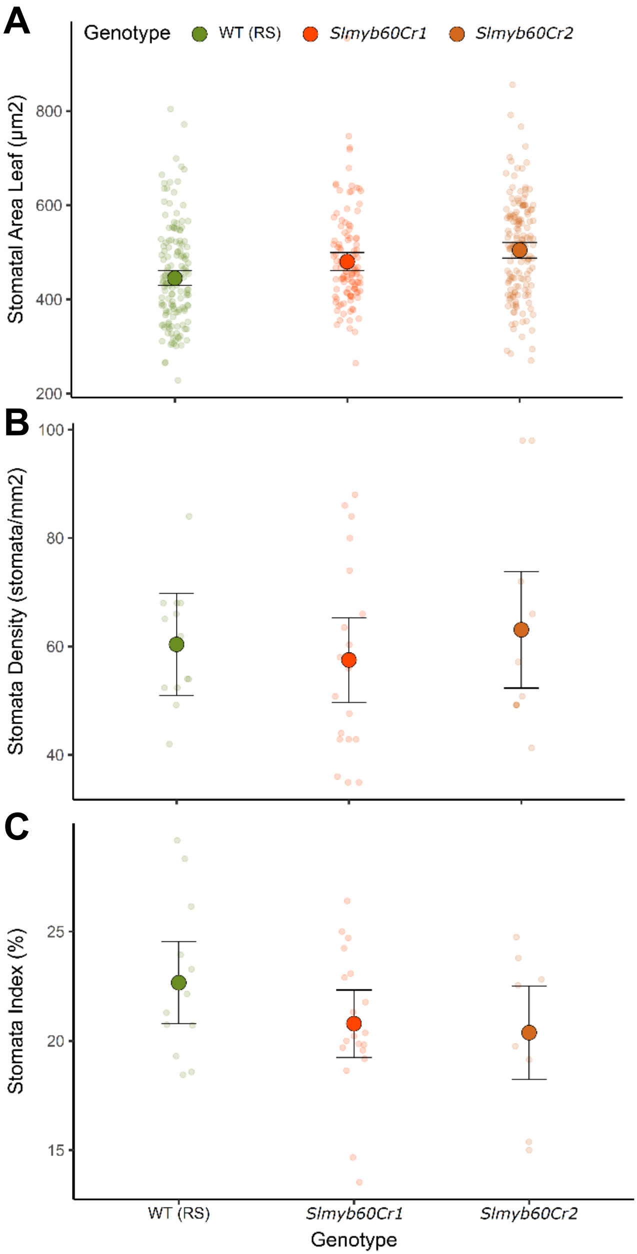

SUPPLEMENTARY FIGURE 3: Analysis of stomatal features in the abaxial side of the wild type (RS) and *slmyb60Cr1* and *-Cr2* lines. Stomatal area was assessed for n = 147, 112, and 154 stomata for wild type, *slmyb60Cr1,* and *-Cr2*, respectively. Stomatal density and stomatal index were measured by counting the number of stomata and epidermal cells on the abaxial leaf surface cleared with 80% EtOH. Five leaves were analyzed for each genotype, for a total of 20 images per line, corresponding to an overall area of approximately 5 mm2. Stomatal density was expressed as the number of stomata per mm2 of epidermal surface. Stomatal index was calculated as: (Number of Stomata x 100) / (Number of Epidermal Cells + Number of Stomata). Genotype only had a statistically significant effect on stomatal area ($p < 0.001$, $F = 12.7$), with stomata always larger in mutants than wild type and this difference being statistically significant both for slmyb60Cr1 ($p < 0.05$, $t = -2.7$) and -Cr2 ($p < 0.001$, $t = -5$) according to a post-hoc test. Large points represent estimated means accompanied by 95% CIs, small points represent raw data.

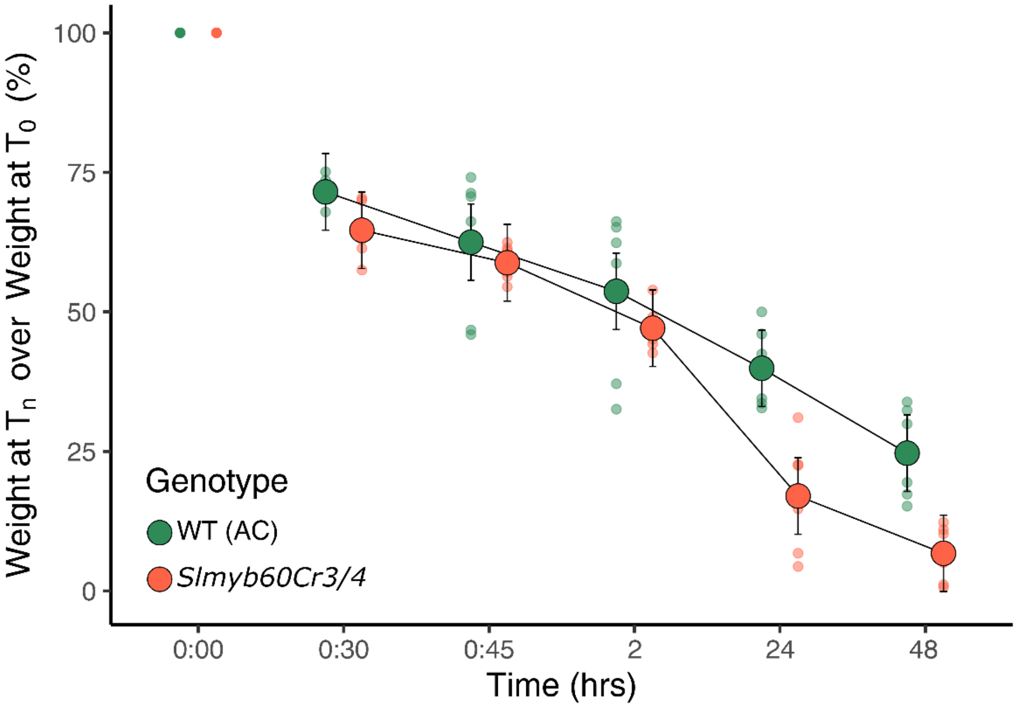

SUPPLEMENTARY FIGURE 4: Time course of water loss from excised leaves (n = 6 for each genotype-timepoint combination) in the wild type (AC) and the *slmyb60Cr3/4* line, expressed as a percentage of the initial fresh weight at the indicated time points. Genotype ($p < 0.001$, Chisq. = 12.2), timepoint ($p < 0.001$, Chisq. = 507.4), and their interaction ($p < 0.01$, Chisq. = 18.2) all had a statistically significant effect. Estimated means of mutants were lower than wild type at all timepoints analyzed, and a post-hoc test showed that this difference was statistically significant at 24 ($p < 0.001$, $t = 4.7$) and 48 hr ($p < 0.001$, $t = 3.7$). Large points represent estimated means accompanied by 95% CIs, small points represent raw data.

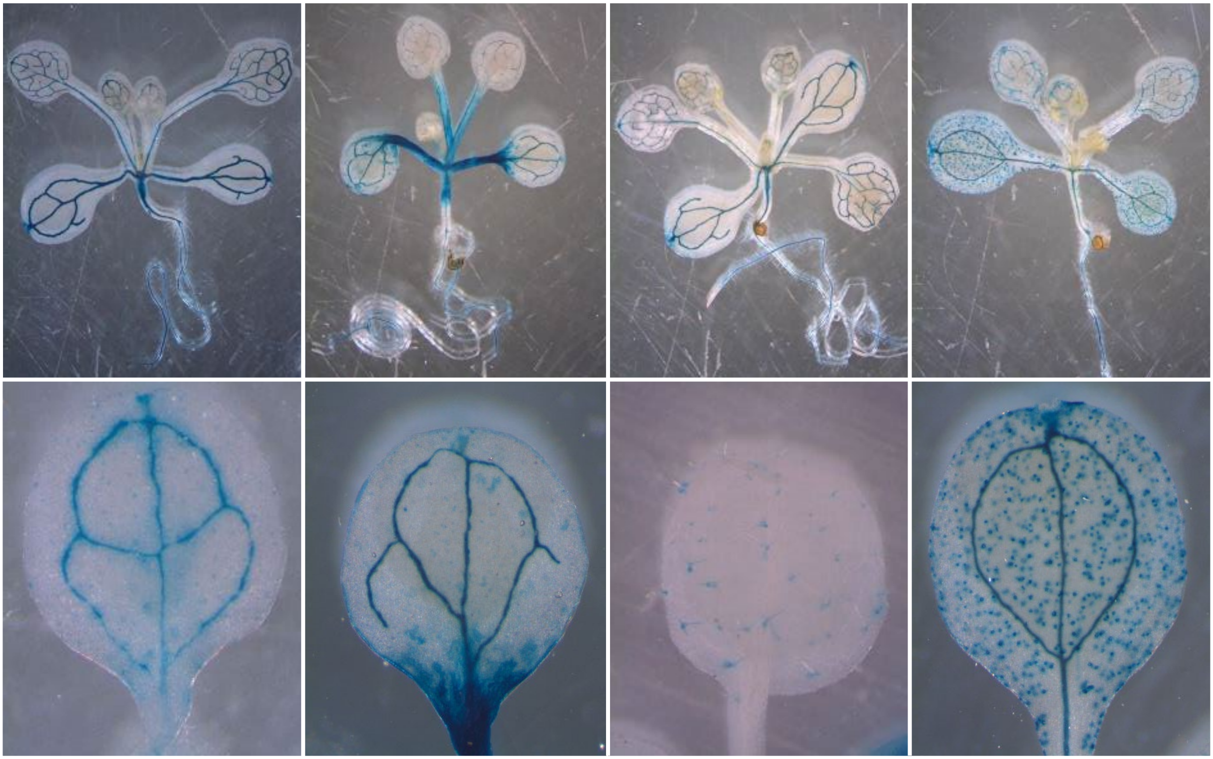

line	cotiledons			leaves				roots	
	Ep.	Vasc.	GCs	Ep.	Vasc.	Trich.	GCs	Ep.	Vasc.
1	+++	+++	-	+	++	++	-	-	+++
2	+++	+++	++	+	+++	-	+	-	+++
3	++	+++	++	-	+	-	+	-	+++
4	+++	+++	-	+	++	++	-	-	+++
5	++	+++	-	+	++	++	-	-	+++
6	-	+++	-	-	+++	-	-	-	+++
7	-	+++	-	-	+++	+++	-	++	+++
8	+	++	-	-	++	+	-	-	++
9	++	+++	-	+	++	++	-	-	+++
10	+++	+++	-	+	++	+	-	-	+++

SUPPLEMENTARY FIGURE 5: GUS expression patterns in seedlings harbouring the *SlMYB60pro:GUS:GFP* construct. GUS assay was performed on 15-day-old plants. The table reports a summary of different GUS profiles observed in independent lines. Ep, epidermis; VASC, vasculature; GC, guard cells; trich., trichomes. +++, strong GUS signal; ++, moderate GUS signal; +, weak GUS signal; -, no GUS signal.

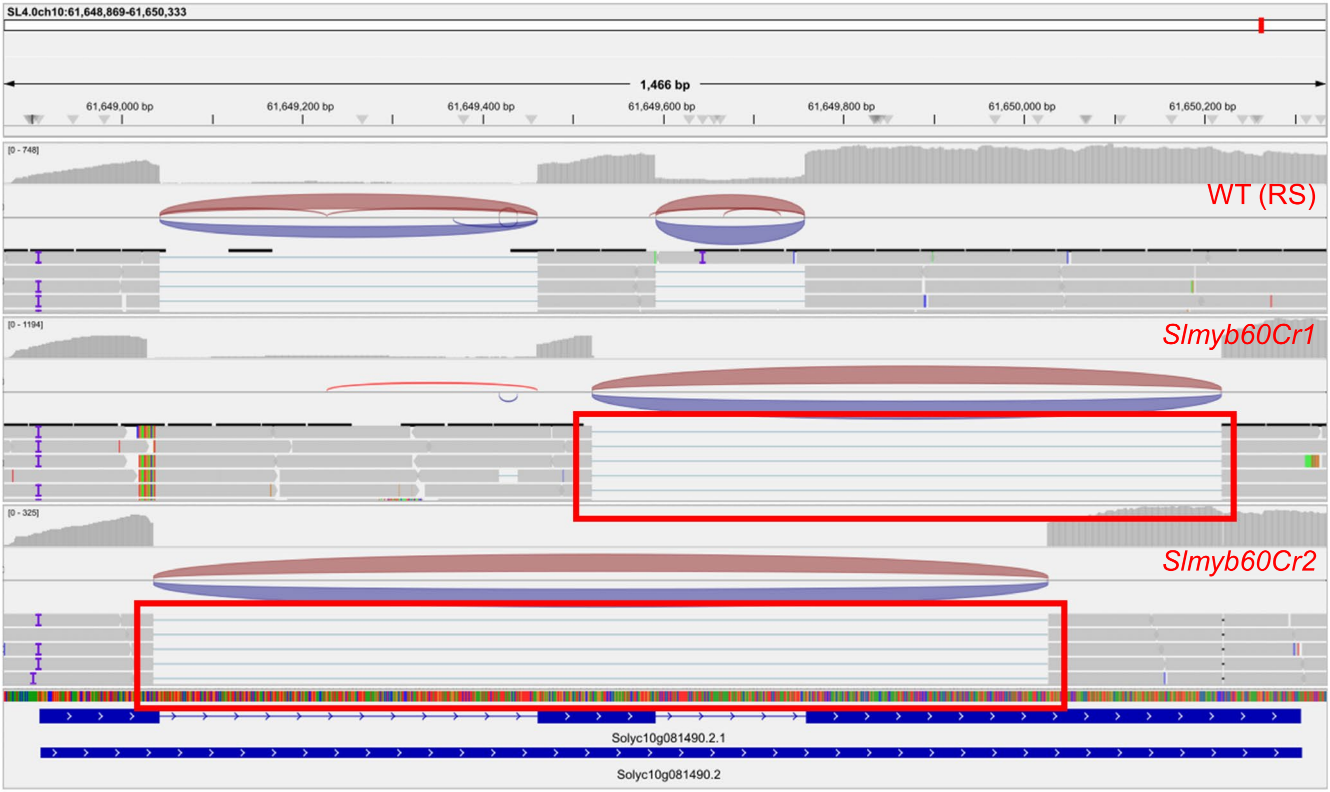

SUPPLEMENTARY FIGURE 6: Snapshot of the Integrative Genome Viewer window software representing the mapped reads on the *Slmyb60* gene (ITAG 4.0 reference) in wild type, *slmyb60Cr1*, and -*Cr2* lines. Differences in spliced reads, indicated by blue (forward reads) and red (reverse reads) arcs, between the wild type and the mutants show different gene structure in the same way as exons reads coverage. At the bottom, the gene structure of Slmyb60 is shown with boxes representing the exons and line the introns. Arrows indicate the gene direction on chromosome.

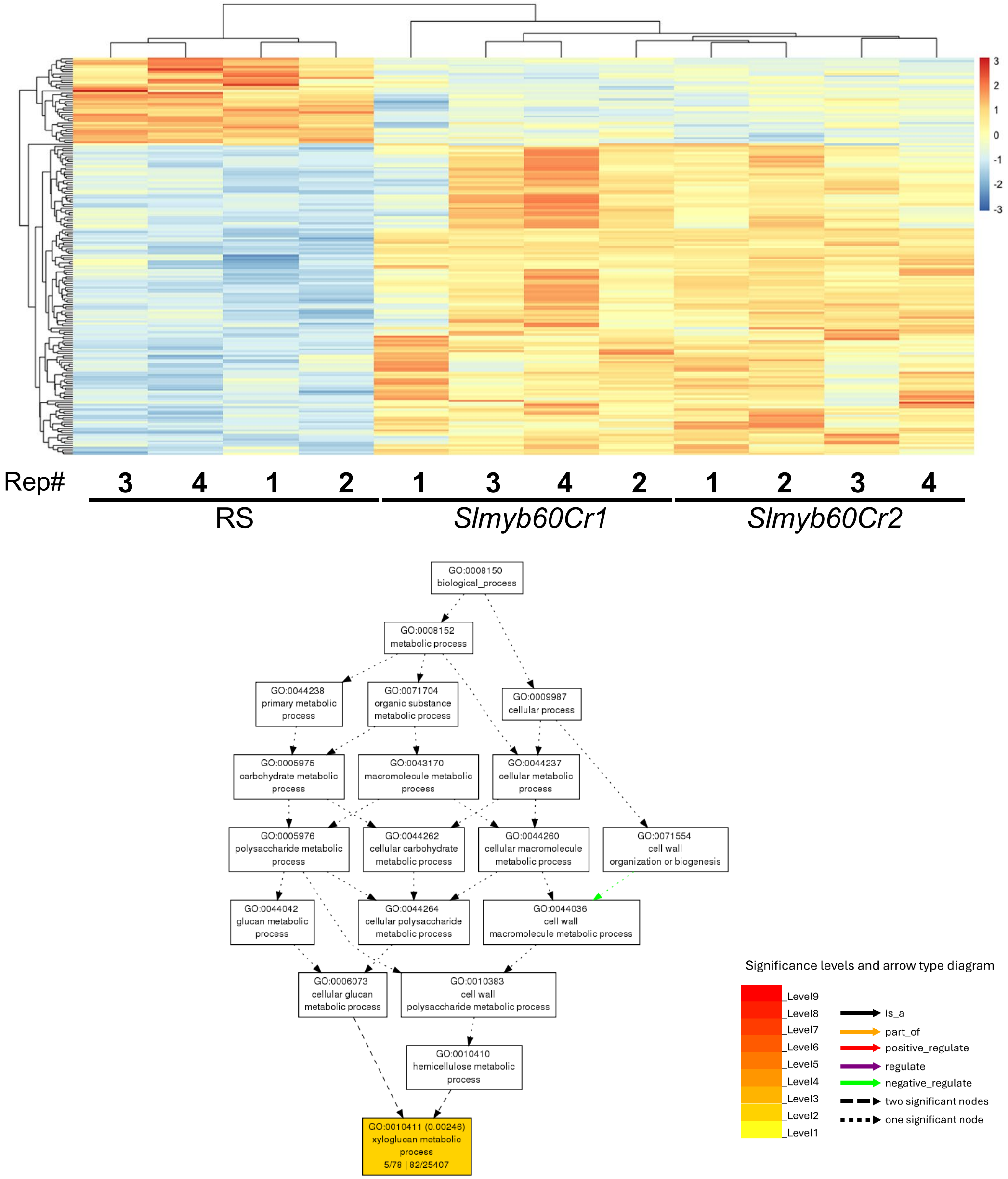

SUPPLEMENTARY FIGURE 7: Heatmap of the common DEGs between the two mutant lines. Numbers 1 to 4 represent the biological replicates. The coloured scale represents the normalized expression value. At the bottom, the top-down hierarchical graphical result of the significant gene ontology term found in the common DEGs performed on AgriGOv2 website.

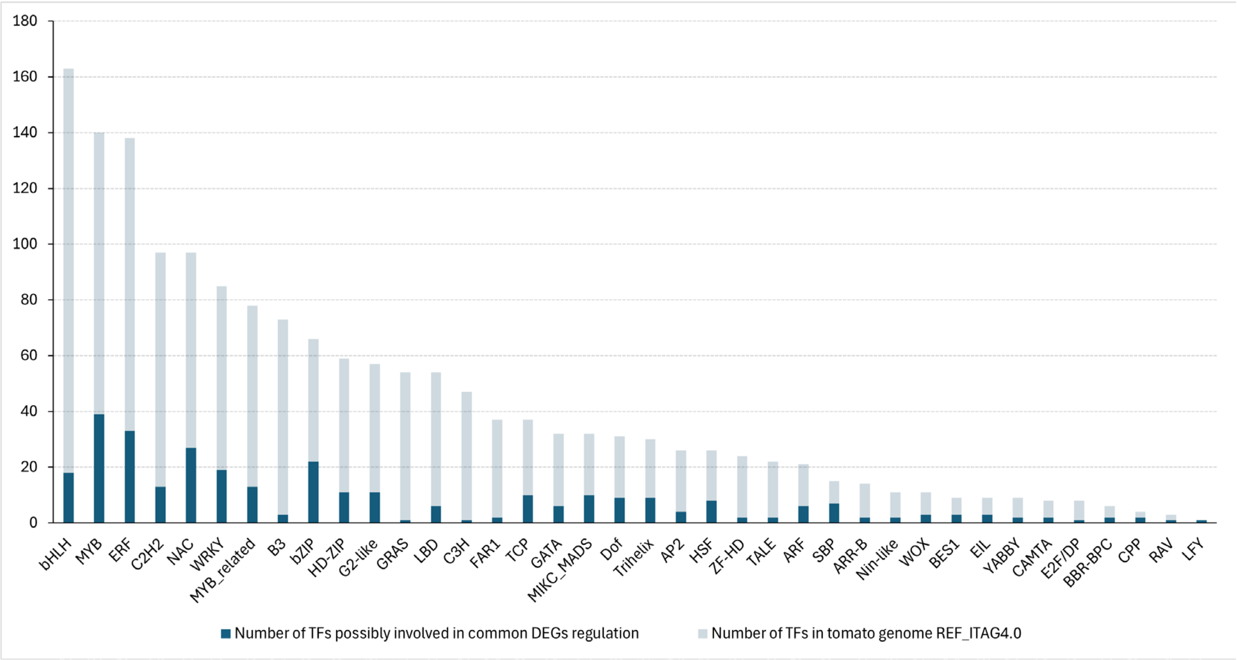

SUPPLEMENTARY FIGURE 8: Histogram of TFs present in the tomato genome reference ITAG4.0 (light blue) and the TFs found to be possibly involved in common DEGs transcriptional regulation (dark blue).

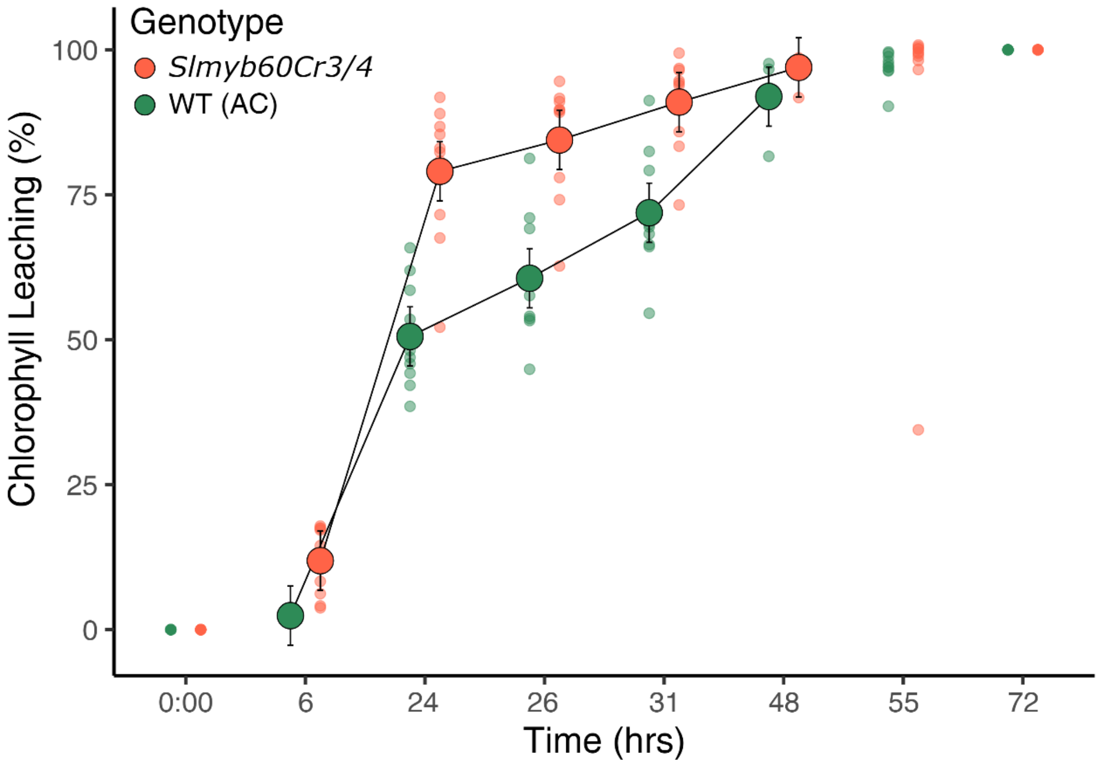

SUPPLEMENTARY FIGURE 9: Chlorophyll leaching assay in leaves excised from wild type AC and *slmyb603/4* edited plants, with n = 10 leaves sampled for each genotype-timepoint combination. Genotype ($p < 0.001$, Chisq. = 30), timepoint ($p < 0.001$, Chisq. = 4296), and their interaction ($p < 0.001$, Chisq. = 90.6) all had a statistically significant effect. Chlorophyll leaching was always estimated as higher in mutants than wild type, and a post-hoc showed these differences to be statistically significant at all timepoints ($p < 0.05$) except the last one. Large points represent estimated means accompanied by 95% CIs, small points represent raw data.

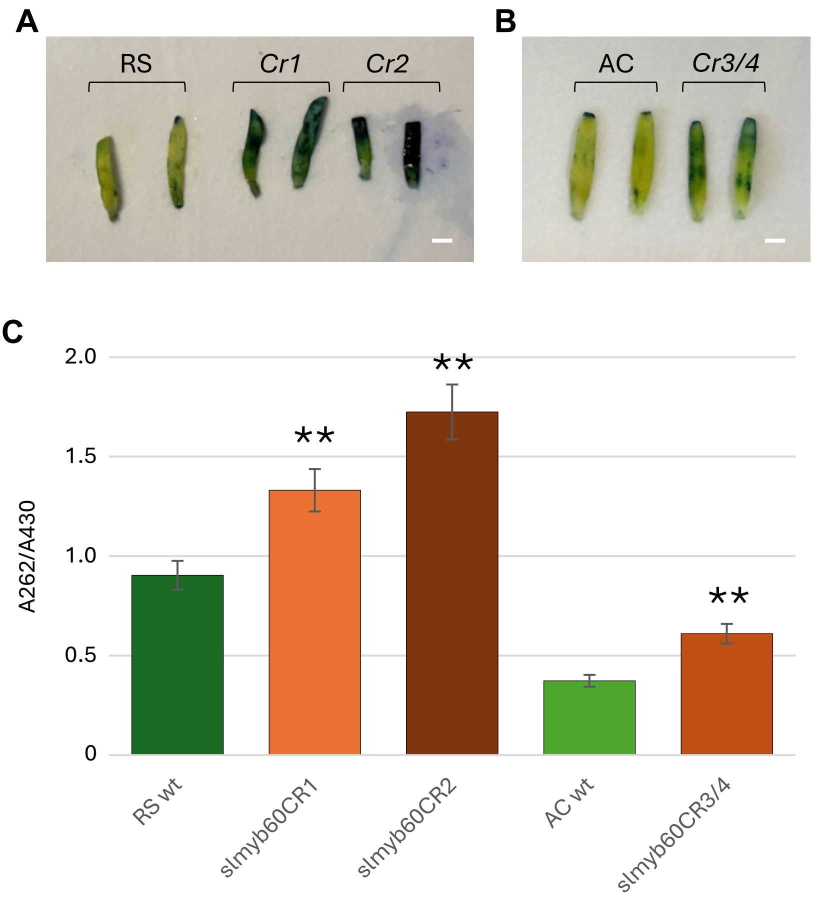

SUPPLEMENTARY FIGURE 10: TB staining of wild type and *slmyb60CR* lines. Increased staining of the slmyb60Cr1 and -2 (A) or of the slmyb603/4 line(B) compared with the respective wild type plants (RS or AC) indicated altered permeability of the cuticle layer. Scale bar: 1 mm. (C) Quantification of the TB staining. Asterisks indicate significant differences compared with the wild type (ANOVA, $p < 0.001$).

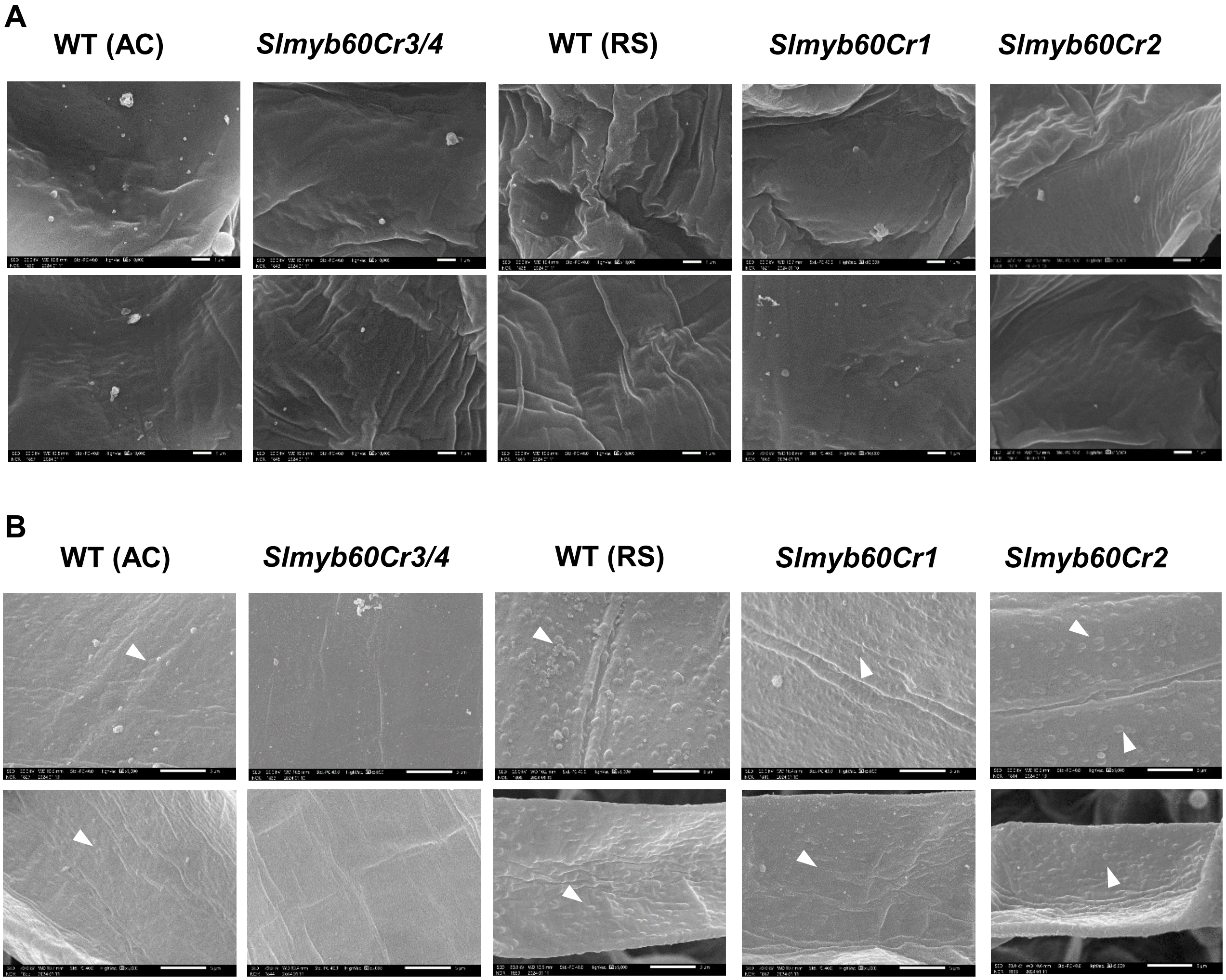

SUPPLEMENTARY FIGURE 11: SEM micrographs of epicuticular wax layer. Pavement cells (A) and non-glandular trichomes (B) on the abaxial and adaxial side of the fully expanded third leaf (terminal leaflet) have been analysed in Ailsa Craig (AC) and Red setter (RS) wild-type (WT) background, *slmyb60Cr3/4, slmyb60Cr1* and *slmyb60Cr2* edited plants. White arrowheads point to cuticle papilla on trichomes (B). Scale bars correspond to 5 µm in the micrographs of trichomes, and to 1 µm for the epidermis.

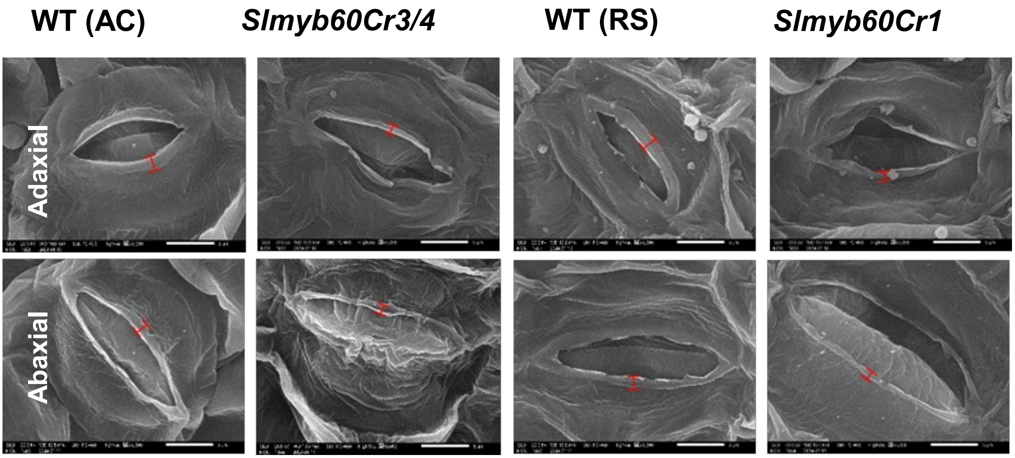

SUPPLEMENTARY FIGURE 12: SEM micrographs of the stomatal outer cuticular ledges (OCLs) in the wild type (AC, RS) and in the *slmyb60Cr3/4* and *slmyb60Cr1* edited lines. Red lines indicate the thickness of OCLs) on the abaxial and adaxial side of fully expanded third leaves (terminal leaflets). Scale bars correspond to 5 µm.

SUPPLEMENTARY TABLE 1: List of guideRNAs and primers used in the study.

CODE	SEQUENCE (5’- 3’)	Target	USE
sg1	AAGTTATATCTGATGTATGG	Guide RNA 1	Gene editing
sg2	TCAGTCCCTACTAATACTGG	Guide RNA 2	Gene editing
sg3	GAGGCCAGGAATCAAAAGGG	Guide RNA 3	Gene editing
sg4	TGAGGCCAGGAATCAAAAGG	Guide RNA 4	Gene editing
sk3	ATGGGGAGGCCACCTTGTT	*SlMYB60*	Sequencing *SlMYB60*
sk4	TTATGTGAATATCATTGGAAGTTCCAT GAG	*SlMYB60*	Sequencing *SlMYB60*
sk18	CTAGAAGTAGTCAAGGCGGC	CmYLCV - TC320	Sequencing plasmid pDIRECT22C
sk32	GGAACCCTAATTCCCTTATCTGG	CmYLCV -TC089R	Sequencing plasmid pDIRECT22C
sk46	AGCTCGGTGGTGATTCAAGG	Cas9 pDIRECT22C	with Sk32 Cas9 presence/absence in transformed tomato
sk47	CGTAGGACGTACGCCAATAGA	*SlMYB60*	Genotyping
sk48	TCGTCCAGATCCTGTCATCA	*SlMYB60*	Genotyping
SlMYB60F1	AAAAAGCAGGCTTGCTACAGTTACCA TTTATT	*SlMYB60*$_{pro}$ with Attb1 site	Cloning *SlMYB60* promoter
SlMYB60R1	AGAAAGCTGGGTGTGTGTGTTATTTT GTTGTA	*SlMYB60*$_{pro}$ with Attb2 site	Cloning *SlMYB60* promoter
p60GTWF1	TATAGAAAAGTTGCACAAGGACACAA GGACATAT	*AtMYB60*$_{pro}$ with Attb1 site	Cloning *AtMYB60* promoter
p60GTWR1	TTTTGTACAAACTTGTCTCTTCCTCTA GATCTCTCTG	*AtMYB60*$_{pro}$ with Attb2 site	Cloning *AtMYB60* promoter

SUPPLEMENTARY TABLE 2: Sequencing and mapping data. Total reads refer to the raw sequening output. Filtered reads refers to the number of reads after trimming. Salmon targets refer tothe number of transcripts used in the Salmon pipeline.

Sample	Total reads	Data (Gbp)	Q20(%)	Q30(%)	GC(%)	Salmon Targets	Filtered reads	mapping rate (%)
WT_rep1	46913988	14.05	99.71	98.39	42.59	33989	39725091	84.67
WT_rep2	41110387	12.31	99.7	98.32	42.8	33989	35338495	85.96
WT_rep3	53145249	15.92	99.67	98.18	42.51	33989	45185791	85.02
WT_rep4	47764149	14.3	99.67	98.17	42.43	33989	40288869	84.35
Cr1_rep1	46109832	13.81	99.69	98.25	42.38	33989	38898539	84.36
Cr1_rep2	41311386	12.37	99.69	98.28	42.28	33989	34558270	83.65
Cr1_rep3	43645276	13.07	99.68	98.25	42.41	33989	36870969	84.48
Cr1_rep4	44708587	13.38	99.68	98.22	42.31	33989	37542771	83.97
Cr2_rep1	47473068	14.2	99.68	98.25	42.29	33989	39659063	83.54
Cr2_rep2	44093855	13.2	99.7	98.32	42.21	33989	36516063	82.81
Cr2_rep3	41436710	12.41	99.7	98.37	42.37	33989	34866030	84.14
Cr2_rep4	45132266	13.52	99.66	98.11	42.26	33989	37859560	83.88

SUPPLEMENTARY TABLE 3: List of common differentially expressed genes (DEGs) in the *slmyb60Cr1* and *slmyb60Cr2* lines compared with the wild type.

ID	CR1_log2(FC)	CR2_log2(FC)	CR1_padj	CR2_padj	Description
Solyc09g015440.1	6,216	4,811	5,596E-09	1,63E-05	GDT1-like protein 3 (AHRD V3.3 *** A0A2G3B0H6_CAPCH)
Solyc01g066790.1	5,734	5,403	4,747E-05	0,0001622	Unknown protein
Solyc04g057840.1	4,827	5,367	0,0035928	0,0008849	Pvr4 (AHRD V3.3 *-* A0A1D5AHY8_CAPAN)
Solyc06g062540.3	4,623	4,308	2,015E-17	2,81E-12	phosphatase (psi14C gene)
Solyc10g050970.1	4,348	3,038	0,0201519	0,0402645	Ethylene Response Factor D.4
Solyc05g025680.3	4,203	3,379	1,092E-09	8,31E-05	Respiratory burst oxidase homolog (AHRD V3.3 *** Q84KK8_NICBE)
Solyc01g108240.3	4,028	3,447	0,0302586	2,27E-05	Ethylene Response Factor D.3
Solyc09g091390.3	3,944	4,183	0,0009006	6,65E-05	glycine-rich protein 5-like (AHRD V3.3 *-* XP_004247821.1)
Solyc02g150135.1	3,716	3,599	1,883E-06	1,59E-07	Unknown protein
Solyc11g011310.3	3,713	3,579	0,0043453	0,0305151	Rhamnogalacturonate lyase family protein (AHRD V3.3 *** A0A2U1PQM1_ARTAN)
Solyc03g093110.3	3,570	1,966	2,182E-05	6,72E-20	Xyloglucan endotransglucosylase/hydrolase (AHRD V3.3 *** Q6RHY0_SOLLC)
Solyc04g081700.3	3,455	3,325	2,02E-211	6,11E-226	Unknown protein
Solyc09g014580.3	3,339	2,309	0,0003003	0,0271692	MLP-like protein 43 (AHRD V3.3 *** A0A2U1L1Z2_ARTAN)
Solyc10g084960.2	3,338	3,243	2,773E-39	3,95E-71	Glutathione S-transferase (AHRD V3.3 *** C0LF68_CAPAN)
Solyc09g064750.2	3,299	3,258	2,004E-89	1,40E-92	Unknown protein
Solyc03g093120.5.1	3,292	1,846	0,0002664	4,91E-18	Xyloglucan endotransglucosylase/hydrolase (AHRD V3.3 *** Q6RHY0_SOLLC)
Solyc06g075520.3	3,225	3,524	1,061E-12	1,23E-43	Glutathione S-transferase (AHRD V3.3 *** A0A200PZB1_9MAGN)
Solyc06g060870.1	3,202	2,880	0,0185435	0,0007149	Phospholipase A1-Ibeta2, chloroplastic (AHRD V3.3 *** A0A2G2ZA78_CAPAN)
Solyc12g008930.3	3,168	2,591	1,07E-05	0,0061855	Basic helix-loop-helix (BHLH) DNA-binding family protein (AHRD V3.3 *-* A0A2U1P678_ARTAN)
Solyc07g056000.2	3,094	1,034	0,0002079	0,0055076	Xyloglucan endotransglucosylase/hydrolase (AHRD V3.3 *** Q43528_SOLLC)
Solyc12g010755.1	3,057	2,024	1,066E-06	0,013083	protein ULTRAPETALA1-like (AHRD V3.3 *** A0A2I4DNL4_9ROSI)
Solyc10g055420.2	2,969	2,401	0,0056095	0,0355204	Unknown protein
Solyc07g042230.1	2,955	3,258	0,033066	0,0052519	Ethylene-responsive transcription factor (AHRD V3.3 *-* A0A2G2WJA8_CAPBA)
Solyc03g093080.3	2,864	1,725	1,156E-06	1,44E-11	Xyloglucan endotransglucosylase/hydrolase (AHRD V3.3 *** Q6RHY0_SOLLC)
Solyc03g093130.3	2,852	2,303	0,0016383	9,45E-17	xyloglucan endotransglucosylase-hydrolase 3
Solyc06g008057.1	2,828	3,135	0,027165	0,007719	Unknown protein
Solyc08g078460.3	2,798	3,391	4,191E-05	1,99E-06	Oxidoreductase family protein (AHRD V3.3 *** A0A2U1N6F1_ARTAN)
Solyc06g076080.3	2,747	1,453	0,0054082	0,0262578	Unknown protein
Solyc08g007820.1	2,692	1,683	0,0006554	0,0015947	Dehydration-responsive element-binding protein 1E (AHRD V3.3 *** A0A2G2W337_CAPBA)
Solyc08g080290.4	2,662	3,134	0,0146456	4,98E-08	Ethylene-responsive transcription factor (AHRD V3.3 *** A0A2G2XKF3_CAPBA)
Solyc11g018774.1	2,659	2,021	0,0327884	0,0009306	Peroxidase (AHRD V3.3 *** M1CN26_SOLTU)
Solyc09g089780.3	2,588	4,025	0,0015874	3,81E-12	2-oxoglutarate (2OG) and Fe(II)-dependent oxygenase superfamily protein (AHRD V3.3 *** O80851_ARATH)
Solyc05g024420.1	2,395	2,257	0,0268759	0,0248827	Unknown protein
Solyc02g077060.2	2,342	1,694	0,0235085	0,0018217	NB-ARC domains-containing protein (AHRD V3.3 *-* A0A2U1QPI4_ARTAN)
Solyc03g123620.4	2,337	1,583	0,0003796	1,75E-20	Pectinesterase (AHRD V3.3 *** A0A2G3D1E1_CAPCH)
Solyc01g087590.3	2,276	2,744	3,504E-07	8,47E-07	Polyamine oxidase (AHRD V3.3 *** Q4H439_TOBAC)
Solyc11g069960.2	2,213	1,892	0,0130363	3,36E-06	RLK-1
Solyc12g077395.1	2,210	2,372	1,347E-05	3,14E-08	Retrovirus-related Pol polyprotein from transposon TNT1-94 (AHRD V3.3 *-* A0A2K3MN99_TRIPR)
Solyc10g008350.4	2,188	1,426	0,0001816	0,0181337	hypothetical protein (AHRD V3.3 --* AT4G28290.2)
Solyc04g005040.1	2,180	1,153	0,0462748	0,0018016	Metalloendoproteinase 1 (AHRD V3.3 *** A0A1J3JDB5_NOCCA)
Solyc03g097050.3	2,153	1,363	8,867E-07	1,83E-12	Cellulose synthase (AHRD V3.3 *** A0A2U1M9E5_ARTAN)
Solyc06g008053.1	2,147	1,866	0,000393	0,0007227	Retrovirus-related Pol polyprotein from transposon TNT1-94 (AHRD V3.3 *-* A0A2K3LF16_TRIPR)
Solyc03g005980.3	2,137	2,164	6,283E-12	5,06E-10	NOD26-like intrinsic protein 1.1
Solyc04g074410.2	2,098	1,322	0,0046313	5,64E-05	Protein EXORDIUM-like 1 (AHRD V3.3 *** A0A2G2ZQI6_CAPAN)
Solyc12g010410.3	2,094	1,724	9,222E-10	2,83E-06	Homeoboxprotein knotted-1-like 3 (AHRD V3.3 *** A0A1U8H944_CAPAN)
Solyc04g074470.1	2,023	2,133	0,0073473	4,14E-07	Protein EXORDIUM-like 2 (AHRD V3.3 *** A0A2G3CNT3_CAPCH)
Solyc12g011023.1	2,011	2,386	0,0002094	2,22E-11	Xyloglucan endotransglucosylase/hydrolase (AHRD V3.3 *** Q6RHX7_SOLLC)
Solyc09g010210.3	1,964	1,708	0,0302586	0,0135199	endo-1,4-beta-glucanase precursor (Cel2)
Solyc01g103790.3	1,923	2,062	0,0263293	0,0050221	Protein kinase superfamily protein (AHRD V3.3 *-* A0A2U1PSD3_ARTAN)
Solyc09g018020.3	1,881	2,488	1,659E-09	6,66E-23	Expansin (AHRD V3.3 *** K4CS69_SOLLC)
Solyc12g049190.3	1,875	1,198	0,0006465	0,0315549	Hcr2-0B (AHRD V3.3 *** Q9ZT09_SOLLC)
Solyc09g010980.1	1,875	1,501	0,0466562	0,0020526	cyclin-dependent kinase inhibitor (AHRD V3.3 -** AT5G02220.1)
Solyc04g074830.1	1,864	1,192	0,0001927	0,0333585	ENTH/ANTH/VHS superfamily protein (AHRD V3.3 *** A0A2U1QGP5_ARTAN)
Solyc09g082870.3	1,857	1,289	0,0177037	6,41E-12	Calcium-transporting ATPase (AHRD V3.3 *** A0A2G2W115_CAPBA)
Solyc10g075090.3	1,841	1,432	3,332E-27	1,90E-06	Non-specific lipid-transfer protein (AHRD V3.3 *** K4D1V1_SOLLC)
Solyc12g057160.1	1,836	1,558	0,0021559	0,0001828	Unknown protein
Solyc09g011860.4	1,809	1,268	0,0221182	1,08E-07	GDP-fucose protein O-fucosyltransferase protein (AHRD V3.3 *** A0A2R6R8C5_ACTCH)
Solyc06g062550.4	1,807	1,729	0,0256945	0,0068402	LES459817 putative phosphatase 14A
Solyc02g087210.3	1,788	1,543	5,093E-05	3,22E-06	Zinc finger AN1 domain-containing stress-associated protein 12 (AHRD V3.3 *** A0A2G2XID2_CAPBA)
Solyc08g008150.1	1,784	1,708	7,069E-07	3,31E-07	Late embryogenesis abundant (LEA) hydroxyproline-rich glycoprotein family (AHRD V3.3 *-* A0A2U1NM32_ARTAN)
Solyc01g057770.3	1,771	1,641	0,0482504	7,86E-11	HCO3-transporter family (AHRD V3.3 *** A0A2U1KUW6_ARTAN)
Solyc02g068680.1	1,770	1,277	0,0019624	0,006027	Cysteine/Histidine-rich C1 domain family protein (AHRD V3.3 *-* Q9FIY8_ARATH)
Solyc12g096790.1	1,764	1,999	0,0266912	0,0133389	HXXXD-type acyl-transferase family protein (AHRD V3.3 *** F4JBC7_ARATH)
Solyc04g076190.1	1,762	1,640	0,0001891	0,0008449	Eukaryotic aspartyl protease family protein (AHRD V3.3 *** F4K5B9_ARATH)
Solyc10g084000.3	1,739	1,314	0,0166722	0,0094808	heavy metal-associated isoprenylated plant protein 3-like (AHRD V3.3 *** A0A1U8EC89_CAPAN)
Solyc02g086700.4	1,732	1,429	0,0226586	0,031338	Glucan endo-1,3-beta-glucosidase (AHRD V3.3 *** A0A2G3D9H7_CAPCH)
Solyc12g096890.1	1,730	1,563	0,0189272	1,17E-05	F-boxprotein (AHRD V3.3 *-* A0A2G3B4N2_CAPCH)
Solyc06g076020.3	1,708	1,131	9,354E-08	1,59E-07	heat shock protein 70 kD
Solyc02g079010.4	1,702	1,170	7,978E-05	0,0012328	Cotton fiber expressed protein (AHRD V3.3 *** A0A1Q9XNG5_GOSHI)
Solyc10g075103.1	1,663	1,696	1,841E-07	3,34E-07	Non-specific lipid-transfer protein (AHRD V3.3 *** K4D1V1_SOLLC)
Solyc10g085700.1	1,656	1,773	0,0051816	0,0119971	Aminotransferase-like, plant mobile domain-containing protein (AHRD V3.3 *-* A0A118K705_CYNCS)
Solyc10g085950.2	1,642	1,566	8,867E-07	1,18E-06	ABC transporter protein (AHRD V3.3 *** A0A1L4BHU2_ORYSI)
Solyc03g083730.1	1,622	1,391	0,0302462	0,0032829	Plant invertase/pectin methylesterase inhibitor superfamily protein, putative (AHRD V3.3 *** A0A061G4U8_THECC)
Solyc06g059840.4	1,609	1,448	3,024E-06	1,06E-05	branched chain alpha-keto acid dehydrogenase E1-alpha subunit

SUPPLEMENTARY TABLE 2: continue.

Solyc01g006950.3	1,592	1,421	3,241E-07	5,21E-27	syntaxin-121-like (AHRD V3.3 *** A0A2I4H4W7_9ROSI)
Solyc11g068620.2	1,583	1,478	0,0004183	1,28E-09	NAC domain (AHRD V3.3 *** A0A200Q6H1_9MAGN)
Solyc07g008930.1	1,577	1,731	0,0189272	0,0080999	DUF4228 domain protein (AHRD V3.3 *** G7JUB6_MEDTR)
Solyc09g089790.3	1,574	1,120	3,177E-12	4,05E-08	2-oxoglutarate (2OG) and Fe(II)-dependent oxygenase superfamily protein (AHRD V3.3 *** O80850_ARATH)
Solyc04g016460.3	1,566	2,203	0,0432588	0,0003545	Unknown protein
Solyc03g098010.3	1,550	1,555	0,0012078	0,0061855	phosphate starvation inducible gene TPSI1
Solyc07g055490.4	1,546	1,232	6,822E-07	9,25E-05	Cytochrome (AHRD V3.3 *** A0A2G2Z466_CAPAN)
Solyc04g056713.1	1,536	1,687	5,699E-35	4,45E-52	Aldehyde dehydrogenase family 2 member mitochondrial-like (AHRD V3.3 *-* A0A2K3PGY0_TRIPR)
Solyc07g008140.3	1,531	1,486	2,917E-12	4,31E-14	Umecyanin (AHRD V3.3 *-* A0A2G2WBI2_CAPBA)
Solyc05g051540.3	1,526	1,240	0,003222	0,0002903	ABC transporter G family member 11 (AHRD V3.3 *-* A0A2G2WTG8_CAPBA)
Solyc12g006970.1	1,517	1,742	0,0056095	9,50E-06	Unknown protein
Solyc06g082440.1	1,514	1,291	5,315E-05	4,11E-05	Non-specific serine/threonine protein kinase (AHRD V3.3 *** G4XMX3_SOLLC)
Solyc11g005860.2	1,494	1,208	0,0076117	0,017631	Aminotransferase (AHRD V3.3 *** A0A200PUW3_9MAGN)
Solyc06g072460.1	1,489	1,424	0,0222138	1,73E-08	Cysteine/Histidine-rich C1 domain family protein (AHRD V3.3 *-* Q9ZUW8_ARATH)
Solyc06g053640.1	1,487	1,509	0,001736	2,68E-05	RING/U-box superfamily protein (AHRD V3.3 *** A0A2U1Q188_ARTAN)
Solyc06g070950.2	1,451	1,370	1,512E-05	0,0028029	ABC transporter A family member 3 (AHRD V3.3 *** A0A2G2ZCF6_CAPAN),Pfam:PF12698
Solyc09g008360.2	1,444	1,583	0,0089369	0,0231986	Heparan-alpha-glucosaminide N-acetyltransferase-like protein (DUF1624) (AHRD V3.3 *** Q94CC1_ARATH)
Solyc12g011030.3	1,438	1,286	0,0029843	0,0082859	xyloglucan endotransglucosylase-hydrolase 9
Solyc09g090990.2	1,433	1,210	0,0035553	0,0160524	Major allergen Pru ar 1 (AHRD V3.3 *** Q5GMN2_CAPCH)
Solyc09g091270.3	1,431	1,247	0,0007779	0,0013911	cotton fiber protein (AHRD V3.3 *** AT5G13880.2)
Solyc01g108360.4	1,421	1,615	0,0021468	0,0009751	Cupredoxin (AHRD V3.3 *** A0A2U1P8C5_ARTAN)
Solyc02g082910.4	1,419	1,249	0,0015824	0,0008849	Acyl-CoA synthetase (AHRD V3.3 *** A0A1Y0KWS9_9PSED)
Solyc04g011480.3	1,417	1,197	6,116E-05	0,0135199	CASP-like protein (AHRD V3.3 *** A0A2G2ZG68_CAPAN)
Solyc04g011990.3	1,407	1,369	0,0004907	0,0001828	Disease resistance protein (AHRD V3.3 *** A0A2U1Q201_ARTAN)
Solyc03g121620.1	1,388	1,052	0,0104779	0,0073606	Late embryogenesis abundant (LEA) hydroxyproline-rich glycoprotein family (AHRD V3.3 *** A0A2U1MMJ0_ARTAN)
Solyc03g113980.3	1,384	1,222	3,749E-06	5,51E-11	Calmodulin binding protein-like (AHRD V3.3 *** A0A200RE12_9MAGN)
Solyc06g050630.3	1,377	1,315	1,682E-05	3,13E-21	Arogenate dehydrogenase 1, chloroplastic (AHRD V3.3 *** A0A2G3C5J6_CAPCH)
Solyc08g081550.4	1,372	1,194	0,0079878	1,37E-05	1-aminocyclopropane-1-carboxylate synthase (AHRD V3.3 *** B2NIX1_CAPCH)
Solyc01g095150.3	1,361	1,069	0,0023165	2,84E-05	late embryogenesis-like protein
Solyc11g071990.2	1,356	1,523	0,0093033	0,0006465	FAD/NAD(P)-binding oxidoreductase family protein (AHRD V3.3 *** A0A2U1M1X0_ARTAN)
Solyc10g044986.1	1,353	1,530	0,0391853	0,0083435	Unknown protein
Solyc05g051530.4	1,341	1,168	0,0028044	0,0010524	ABC transporter G family member 11 (AHRD V3.3 *** A0A2G2ZJ77_CAPAN)
Solyc01g098690.2	1,322	1,038	0,0018353	0,0011282	Receptor-like protein 12 (AHRD V3.3 *** A0A1J6JWI2_NICAT)
Solyc10g074540.1	1,319	1,334	0,0266912	7,92E-14	Protein EXORDIUM-like 3 (AHRD V3.3 *** A0A1U8DVK9_CAPAN)
Solyc09g014610.4	1,318	1,274	0,0416767	0,0460354	S-type anion channel SLAH3 (AHRD V3.3 *** A0A2G3BJK2_CAPCH)
Solyc06g070960.3	1,299	1,279	0,0020788	0,0023427	ABC transporter A family member 2 (AHRD V3.3 *** A0A2G3AUS5_CAPCH)
Solyc06g074620.3	1,296	1,031	0,0008665	0,0143402	hypothetical protein (AHRD V3.3 *** AT2G27830.1)
Solyc06g030470.4	1,270	1,128	1,828E-06	2,61E-13	UPSTREAM OF FLC protein (DUF966) (AHRD V3.3 *** Q8GYT8_ARATH)
Solyc04g077470.3	1,267	1,253	0,0024544	1,27E-26	Cellulose synthase-like C4, glycosyltransferase family 2 (AHRD V3.3 *** A9TYJ5_PHYPA)
Solyc02g080040.4	1,263	1,066	0,0011153	4,80E-11	Cysteine-rich receptor-like protein kinase 29 (AHRD V3.3 *** A0A2G2XFA8_CAPBA)
Solyc02g065210.4	1,263	1,034	0,0022839	0,0277059	Cytochrome P450 (AHRD V3.3 *** A0A1U9X5Y9_THAGA)
Solyc05g056400.3	1,242	1,371	0,0004808	3,40E-09	Protein disulfide-isomerase (AHRD V3.3 *** A0A2G3AZ54_CAPCH)
Solyc06g074360.4	1,238	1,013	0,0420335	0,0297236	Zinc finger protein WIP3 (AHRD V3.3 *** A0A2G2ZDE0_CAPAN)
Solyc10g081570.3	1,237	1,856	0,0005549	1,05E-07	Marmande
Solyc12g100240.1	1,218	1,330	0,0266912	0,0373924	Fatty acid desaturase (AHRD V3.3 *-* A0A200QRI1_9MAGN)
Solyc06g064900.3	1,215	1,008	2,759E-05	0,0001274	Unknown protein
Solyc07g052790.3	1,210	1,667	0,0168044	0,0049085	Disease resistance protein (TIR-NBS-LRR class) (AHRD V3.3 *** F4KIC7_ARATH)
Solyc01g006320.4	1,190	1,515	0,0096615	1,01E-07	Late embryogenesis abundant (LEA) hydroxyproline-rich glycoprotein family (AHRD V3.3 *** Q9LJT9_ARATH)
Solyc01g103470.2	1,188	1,111	0,0330241	3,65E-05	proline-rich receptor-like protein kinase PERK4 (AHRD V3.3 --* XP_026410247.1)
Solyc02g080050.1	1,184	1,031	0,0046502	0,0002717	Cysteine-rich receptor-like protein kinase (AHRD V3.3 *** A0A2K3NTV2_TRIPR)
Solyc07g056210.4	1,182	1,142	0,0019943	0,0024651	Heavy metal-associated isoprenylated plant protein 47 (AHRD V3.3 *-* A0A2P4MV36_QUESU)
Solyc00g500406.1	1,182	1,649	0,042063	0,0008464	Disease resistance protein (TIR-NBS-LRR class) (AHRD V3.3 *-* A0A2K3PDT8_TRIPR)
Solyc06g060360.3	1,182	1,077	0,0051976	0,0007544	Adenine nucleotide alpha hydrolases-like superfamily protein (AHRD V3.3 *** A0A1P8APG6_ARATH)
Solyc02g094400.4	1,170	1,246	6,283E-12	5,61E-08	Glycerophosphodiester phosphodiesterase GDPD2 (AHRD V3.3 *** A0A2G3DA15_CAPCH)
Solyc04g072070.3	1,162	1,503	0,0027882	4,34E-08	WRKY transcription factor 55
Solyc01g112000.4	1,132	1,055	0,0002094	1,89E-31	expansin-like protein precursor 1
Solyc12g099465.1	1,124	1,062	0,0137271	1,32E-07	senescence-associated gene 21 (AHRD V3.3 -** AT4G02380.1)
Solyc12g006980.2	1,121	1,253	0,0057967	2,07E-26	Leucine-rich repeat family protein (AHRD V3.3 *-* A0A061GNA0_THECC)
Solyc11g069220.2	1,116	1,066	0,0177375	0,0057373	MLO-like protein (AHRD V3.3 *** K4DA78_SOLLC)
Solyc08g080530.1	1,111	1,514	0,0168044	0,0376366	C2 calcium-dependent membrane targeting (AHRD V3.3 *** A0A2U1N7N9_ARTAN)
Solyc03g093890.3	1,098	1,146	0,0159169	3,11E-05	R2R3MYB transcription factor 52
Solyc06g069740.1	1,090	1,136	0,0337217	0,0092384	Calcium-binding EF-hand family protein (AHRD V3.3 *** A0A2U1NCV0_ARTAN)
Solyc03g033790.4	1,075	1,081	3,041E-06	0,0002571	P-loop containing nucleoside triphosphate hydrolases superfamily protein (AHRD V3.3 *** A0A2U1NGS8_ARTAN)
Solyc09g092520.3	1,073	1,958	0,0346075	2,72E-38	xyloglucan endotransglycosylase
Solyc02g064680.4	1,064	1,335	0,002294	8,27E-17	Calcium-transporting ATPase (AHRD V3.3 *** A0A1U8FI07_CAPAN)
Solyc12g011200.3	1,063	1,212	0,0001361	1,90E-06	WRKY transcription factor 28
Solyc07g044960.1	1,051	1,101	0,0046747	1,24E-05	Xyloglucan galactosyltransferase KATAMARI1 (AHRD V3.3 *** A0A2K3L4H3_TRIPR)
Solyc01g103590.4	1,048	1,339	0,019763	0,0001482	Lactoylglutathione lyase / glyoxalase (AHRD V3.3 *** A0A1Y1HY85_KLENI)
Solyc05g056380.3	1,034	1,214	0,0030187	0,0001021	patatin-like phospholipase domain protein (AHRD V3.3 *** AT1G76980.2)
Solyc02g090360.3	1,029	1,371	0,0063735	1,84E-06	L-ascorbate oxidase-like protein (AHRD V3.3 *** A0A1U8FPI3_CAPAN)
Solyc10g079380.2	1,010	1,502	0,0317222	0,0008614	Heat stress transcription factor B-3 (AHRD V3.3 *-* A0A2G2VW77_CAPBA)
Solyc12g096960.2	1,004	1,032	0,0396076	0,0274405	Major allergen Pru ar 1 (AHRD V3.3 *** Q5GMN2_CAPCH)
Solyc08g007430.2	-1,021	-1,532	0,0025294	1,94E-08	NIT2
Solyc08g077170.3	-1,034	-1,583	0,0005106	1,45E-09	Protein NRT1 / PTR FAMILY 7.3 (AHRD V3.3 *** A0A1U8GJT1_CAPAN)
Solyc07g052700.3	-1,039	-1,105	0,0010283	3,94E-05	MADS-box transcription factor (AHRD V3.3 *** A0A2K3PNN3_TRIPR)

SUPPLEMENTARY Table 2: continue.

Solyc02g088320.4	-1,073	-1,605	0,0122347	3,99E-05	Protein ENHANCED DISEASE RESISTANCE 2-like (AHRD V3.3 *** A0A1J3E5X3_NOCCA)
Solyc02g077590.1	-1,093	-1,595	0,040396	3,44E-07	Homeobox-leucine zipper-like protein (AHRD V3.3 *-* Q3HRT1_PICGL)
Solyc12g035190.2	-1,201	-1,002	0,0003966	7,57E-05	Anthranilate phosphoribosyltransferase (AHRD V3.3 *** A0A2U1N166_ARTAN)
Solyc01g094750.4	-1,225	-1,306	0,0003711	2,67E-10	Cytochrome P450 (AHRD V3.3 *** A0A2U1NG86_ARTAN)
Solyc06g008560.2	-1,231	-1,244	0,0009779	0,0001044	ternary complex factor MIP1 leucine-zipper protein (Protein of unknown function%2C DUF547) (AHRD V3.3 *** AT2G39690.1)
Solyc09g010510.4	-1,283	-1,069	0,0001754	0,0015476	NAD(P)-binding Rossmann-fold superfamily protein (AHRD V3.3 *** A0A2U1QM80_ARTAN)
Solyc11g006230.3	-1,315	-1,152	0,0013544	1,79E-06	GRF1-interacting factor 1 (AHRD V3.3 *-* A0A2G2Y6I7_CAPAN)
Solyc09g008760.1	-1,367	-1,773	0,0013469	2,92E-06	VQ motif-containing protein 29 (AHRD V3.3 *** A0A2G3BIN0_CAPCH)
Solyc03g120920.1	-1,381	-2,400	1,017E-08	3,04E-18	DUF1677 domain-containing protein (AHRD V3.3 *** A0A1Q3DB84_CEPFO)
Solyc06g071500.3	-1,458	-1,384	2,415E-10	3,53E-14	Boron transporter 1 (AHRD V3.3 *** A0A2G2WM36_CAPBA)
Solyc06g076930.2	-1,467	-2,325	2,592E-05	1,47E-10	ABC transporter-like (AHRD V3.3 *** A0A200PWT6_9MAGN)
Solyc07g066180.2	-1,548	-1,136	5,495E-09	1,98E-21	P-loop containing nucleoside triphosphate hydrolases superfamily protein (AHRD V3.3 *** AT5G56220.2)
Solyc06g053730.1	-1,591	-1,876	1,67E-12	3,16E-30	Serine/threonine-protein kinase BLUS1 (AHRD V3.3 *** A0A2G3C6N8_CAPCH)
Solyc05g007010.3	-1,718	-1,672	7,294E-06	7,64E-08	Glyoxal oxidase (AHRD V3.3 *** A0A200PQ20_9MAGN)
Solyc06g009770.1	-1,721	-1,432	0,000215	2,09E-05	hypothetical protein (AHRD V3.3 *** AT3G62650.2)
Solyc09g090090.2	-1,812	-2,242	0,0002061	0,0002185	Phosphoenolpyruvate carboxylase kinase 1 (AHRD V3.3 *** A0A2G2XZR5_CAPAN)
Solyc06g062460.3	-1,976	-1,310	3,242E-07	0,0053093	bHLH transcription factor136
Solyc04g007000.2	-2,056	-1,838	1,828E-06	1,06E-05	AP2/B3 transcription factor family protein (AHRD V3.3 *** A0A2U1MEM3_ARTAN)
Solyc12g036470.2	-2,102	-1,100	2,415E-12	0,0029879	bHLH transcription factor 071
Solyc05g052680.1	-2,124	-1,939	1,241E-11	1,56E-08	HXXXD-type acyl-transferase family protein (AHRD V3.3 *** V5PZN5_9CARY)
Solyc11g006710.2	-2,236	-2,353	0,0063351	7,84E-06	Cationic amino acid transporter (AHRD V3.3 *** S8DTC6_9LAMI)
Solyc02g063140.4	-2,243	-2,623	2,824E-07	6,73E-09	3-ketoacyl-CoA synthase (AHRD V3.3 *** A0A2G3D1W0_CAPCH)
Solyc11g032050.2	-2,363	-1,970	1,205E-05	2,67E-05	GDSL esterase/lipase (AHRD V3.3 *** A0A2G3CP09_CAPCH)
Solyc09g008750.1	-2,399	-1,367	8,017E-14	4,55E-06	VQ motif-containing protein 29 (AHRD V3.3 *** A0A2G2VXX9_CAPBA)
Solyc10g018190.2	-2,690	-4,673	0,000139	5,28E-10	2-oxoglutarate (2OG) and Fe(II)-dependent oxygenase superfamily protein (AHRD V3.3 *** A0A2U1NR61_ARTAN)
Solyc11g005980.3	-2,716	-2,341	0,000291	0,0023674	Callose synthase (AHRD V3.3 *** K7PRK8_MAIZE)
Solyc11g011980.3	-2,764	-2,926	9,886E-10	4,03E-15	WD40 repeat (AHRD V3.3 *** A0A200RBE1_9MAGN)
Solyc11g061755.1	-2,808	-3,505	0,0073473	0,0015964	Retrovirus-related Pol polyprotein from transposon TNT 1-94 (AHRD V3.3 *-* A0A2G3D5C8_CAPCH)
Solyc10g081490.2	-2,986	-2,742	1,329E-23	6,67E-26	R2R3MYB transcription factor 60
Solyc05g052650.2	-3,244	-2,397	0,0004839	0,000562	HXXXD-type acyl-transferase family protein (AHRD V3.3 *-* V5PZN5_9CARY)
Solyc02g088430.1	-3,385	-4,430	0,0066867	0,0024092	ATP-dependent DNA helicase (AHRD V3.3 *-* A0A2G2XW06_CAPAN)
Solyc03g005900.4	-3,495	-2,717	9,651E-33	7,62E-24	GDSL esterase/lipase (AHRD V3.3 *** A0A2G2XF69_CAPBA)
Solyc10g054900.2	-3,689	-5,233	0,0001272	1,05E-06	Proline-rich protein (AHRD V3.3 *** Q9M6T7_NICGL)
Solyc10g047110.2	-3,874	-5,239	0,0050698	0,0002571	Peroxidase (AHRD V3.3 *** K4CZX5_SOLLC)
Solyc11g150135.1	-4,910	-4,965	0,0011848	0,0004195	Retrovirus-related Pol polyprotein from transposon TNT 1-94 (AHRD V3.3 *-* A0A2G3D5C8_CAPCH)
Solyc12g062200.1	-6,136	-6,181	0,0002612	8,81E-05	Unknown protein
Solyc08g006280.2	-6,657	-6,696	0,0009896	0,0003847	Transcriptional factor B3 family protein (AHRD V3.3 *-* A0A1P8AYQ9_ARATH)
